# Historical shifting in grain mineral density of landmark rice and wheat cultivars released over the past 50 years in India

**DOI:** 10.1038/s41598-023-48488-5

**Published:** 2023-11-30

**Authors:** Sovan Debnath, Ahana Dey, Rubina Khanam, Susmit Saha, Dibyendu Sarkar, Jayanta K. Saha, Mounissamy V. Coumar, Bhaskar C. Patra, Tufleuddin Biswas, Mrinmoy Ray, Madhari S. Radhika, Biswapati Mandal

**Affiliations:** 1 Directorate of Research, Bidhan Chandra Krishi Viswavidyalaya, Kalyani, West Bengal 741 235 India; 2 Department of Agricultural Chemistry and Soil Science, Faculty of Agriculture, Bidhan Chandra Krishi Viswavidyalaya, Mohanpur, West Bengal 741 252 India; 3 Indian Council of Agricultural Research (ICAR)-Central Institute of Temperate Horticulture, Regional Station Mukteshwar, Nainital, Uttarakhand 263 138 India; 4 ICAR-National Rice Research Institute, Cuttack, Odisha 753 006 India; 5 College of Agriculture, Bidhan Chandra Krishi Viswavidyalaya, Burdwan Sadar, West Bengal 713 101 India; 6 Division of Environmental Soil Science, ICAR-Indian Institute of Soil Science, Bhopal, Madhya Pradesh 462 038 India; 7 Department of Agricultural Statistics, Faculty of Agriculture, Bidhan Chandra Krishi Viswavidyalaya, Mohanpur, West Bengal 741 252 India; 8 Department of Agricultural Economics and Statistics, M.S. Swaminathan School of Agriculture, Centurion University of Technology and Management, Bhubaneswar, Odisha 761 211 India; 9 Division of Forecasting and Agricultural Systems Modeling, ICAR-Indian Agricultural Statistics Research Institute, New Delhi, 110 012 India; 10 Department of Dietetics, Indian Council of Medical Research-National Institute of Nutrition, Hyderabad, Telangana 500 007 India; 11 Present Address: ICAR-Central Agroforestry Research Institute, Jhansi, Uttar Pradesh 284 003 India

**Keywords:** Metals, Sustainability, Public health, Plant sciences, Agroecology

## Abstract

The ‘Green Revolution (GR)’ has been successful in meeting food sufficiency in India, but compromising its nutritional security. In a first, we report altered grain nutrients profile of modern-bred rice and wheat cultivars diminishing their mineral dietary significance to the Indian population. To substantiate, we evaluated grain nutrients profile of historical landmark high-yielding cultivars of rice and wheat released in succeeding decades since the GR and its impacts on mineral diet quality and human health, with a prediction for decades ahead. Analysis of grain nutrients profile shows a downward trend in concentrations of essential and beneficial elements, but an upward in toxic elements in past 50 y in both rice and wheat. For example, zinc (Zn) and iron (Fe) concentration in grains of rice decreased by ~ 33.0 (*P* < 0.001) and 27.0% (*P* < 0.0001); while for wheat it decreased by ~ 30.0 (*P* < 0.0001) and 19.0% (*P* < 0.0001) in past more than 50 y, respectively. A proposed mineral-diet quality index (M-DQI) significantly (*P* < 0.0001) decreased ~ 57.0 and 36.0% in the reported time span (1960–2010) in rice and wheat, respectively. The impoverished M-DQI could impose hostile effects on non-communicable diseases (NCDs) like iron-deficiency anemia, respiratory, cardiovascular, and musculoskeletal among the Indian population by 2040. Our research calls for an urgency of grain nutrients profiling before releasing a cultivar of staples like rice and wheat in the future.

## Introduction

Cereals, mostly rice and wheat, are the foundation of food and nutritional security for the humanity^1,2^, particularly of the people of India, providing > 50% of their daily energy requirements. Although a large number of high-yielding cultivars (HYVs) of rice and wheat have been released in the past 50 y since the ‘Green Revolution (GR)’ in India^3^, a few of them are considered as landmark cultivars. These cultivars showed wider adaptability and resistant to various biotic and abiotic stresses across or within the agro-climatic zones of the country, and thus widely adopted by the farmers. Landmark cultivars eventually transformed fortunes of the cereal growers and supported the GR vis-à-vis ensuring food sufficiency of the country.

Despite food sufficiency, it is quite surprising that problem of undernutrition still prevalent in India^4,5^, putting a huge economic burden on its society^6,7^. Of the 768.0 million undernourished people of the world (9.8%)^8^, India is the home of 224.3 million (2.9%)^8,9^. At the heart of this problem are the food systems which are rigidly focused on maximization of yields and economic value^10^, without much consideration for the food value; particularly total mineral elements content, known as the ionome and its impacts on the human health. Also, the problem is possibly aggravated by the reduced consumption of low yielding nutrient-dense coarse cereals (e.g., millets and sorghum)^4^, orphaned during GR, by the large section of Indian society^11^. Nevertheless, Government of India’s policy to resurrect the orphan coarse cereals, through genetic biofortification program (such as Fe- and Zn-pearl millet, finger millet)^12–14^, to the consumers is a commendable effort to alleviate micronutrient deficiencies (MND) in vulnerable Indian population. But the consumers’ preference to cereals, in recent past, remains highly skewed towards rice and wheat over the coarse cereals in India, making them chief staples.

The ‘ionome’ represents mineral nutrients and trace elements composition of an organism relevant to biological or environmental significance^15,16^. A few preliminary reports have shown a declining trend in grain loading of essential minerals in the modern cereal cultivars^17–20^, out of genetic exclusion of traits related to enhancing grain mineral loading^21,22^. This could jeopardize lives of millions of people subsisting on cereal-diets since cereals are also inheritably poor in mineral elements and their bioavailability^23,24^. Recently, we reported such a phenomenon in Indian cultivars also and apprehended its manifestations on human health with prevalence of a few disorders, particularly MNDs in Indian population^25^. Contrarily, a perception has also been grown that modern cereal breeding has increased toxic element components of the grains^26^. Ordinarily, such observations are reasoned as a mere manifestation of low mineral phyto-availability in soils^27^ or soil contamination with toxic elements^28^, due to intensive farming and industrialization.

Fan et al.^18^ and recently Debnath et al.^25^ reported that decrease in grain minerals density in cereals was not related with their depletion in the soils. It possibly hints out of a disruption in crop plants’ inherent intricate regulatory mechanisms for balanced uptake and distribution of mineral nutrients inadvertently created in course of the past breeding programs. In the present study, we tried to unearth the existence and the extent, if there be any, of such a problem of altered grain mineral content with widely adopted (each at least > 5.0 m ha) sixteen (16) and eighteen (18) landmark high-yielding cultivars of rice and wheat, respectively in India released decade-wise during the past 50 y since the beginning of green revolution in 1960s. We further assessed the possible impacts of the existence of such an altered grain mineral content on mineral diet quality (MDQ) of their grains and its manifestations on human health with prediction for a near future.

## Results

### Analyzing trend in grain nutrient concentration in landmark cultivars

#### Essential elements

**Figure 1:**
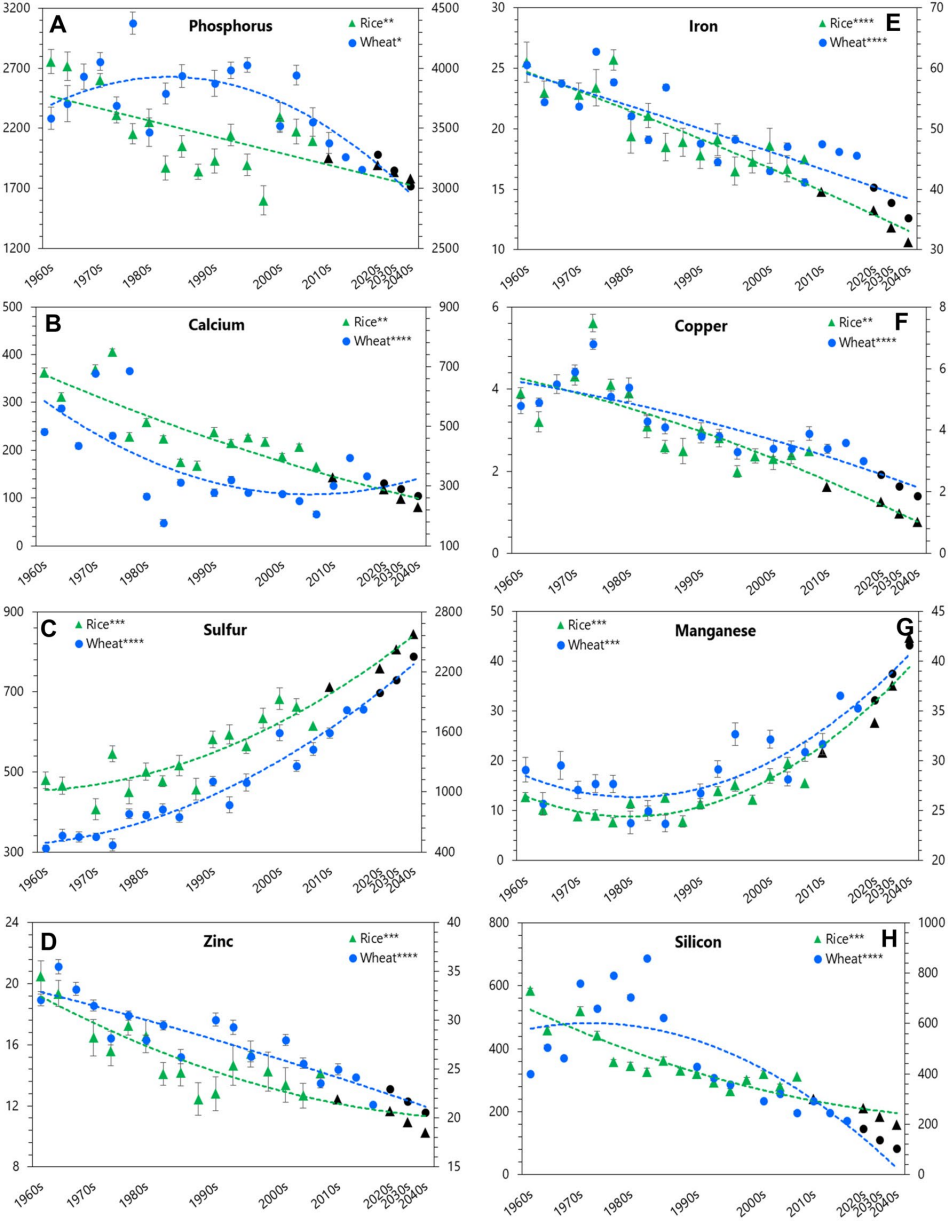

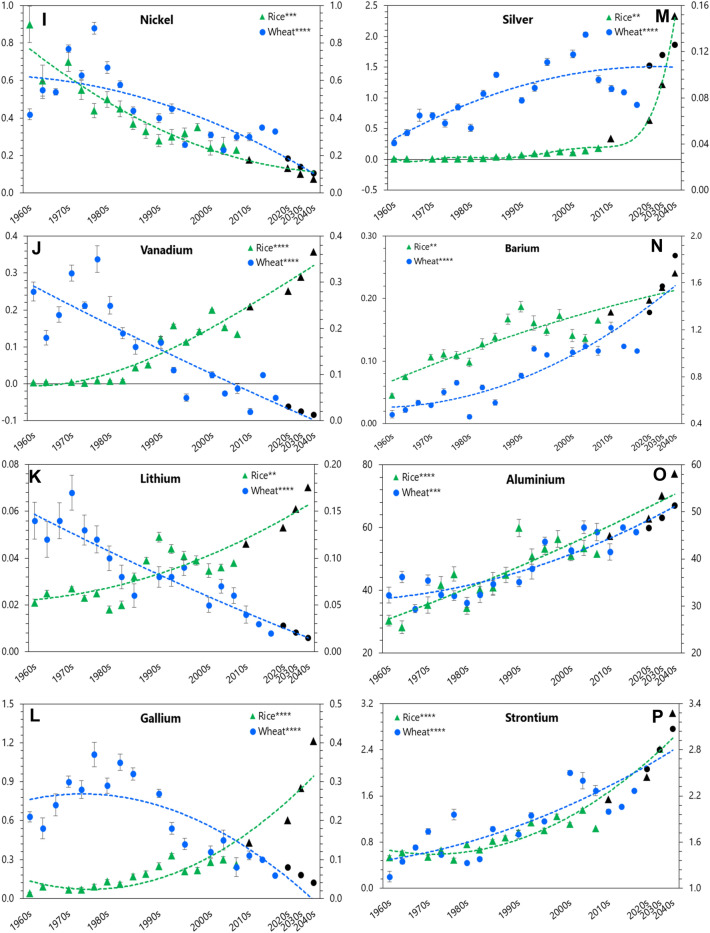

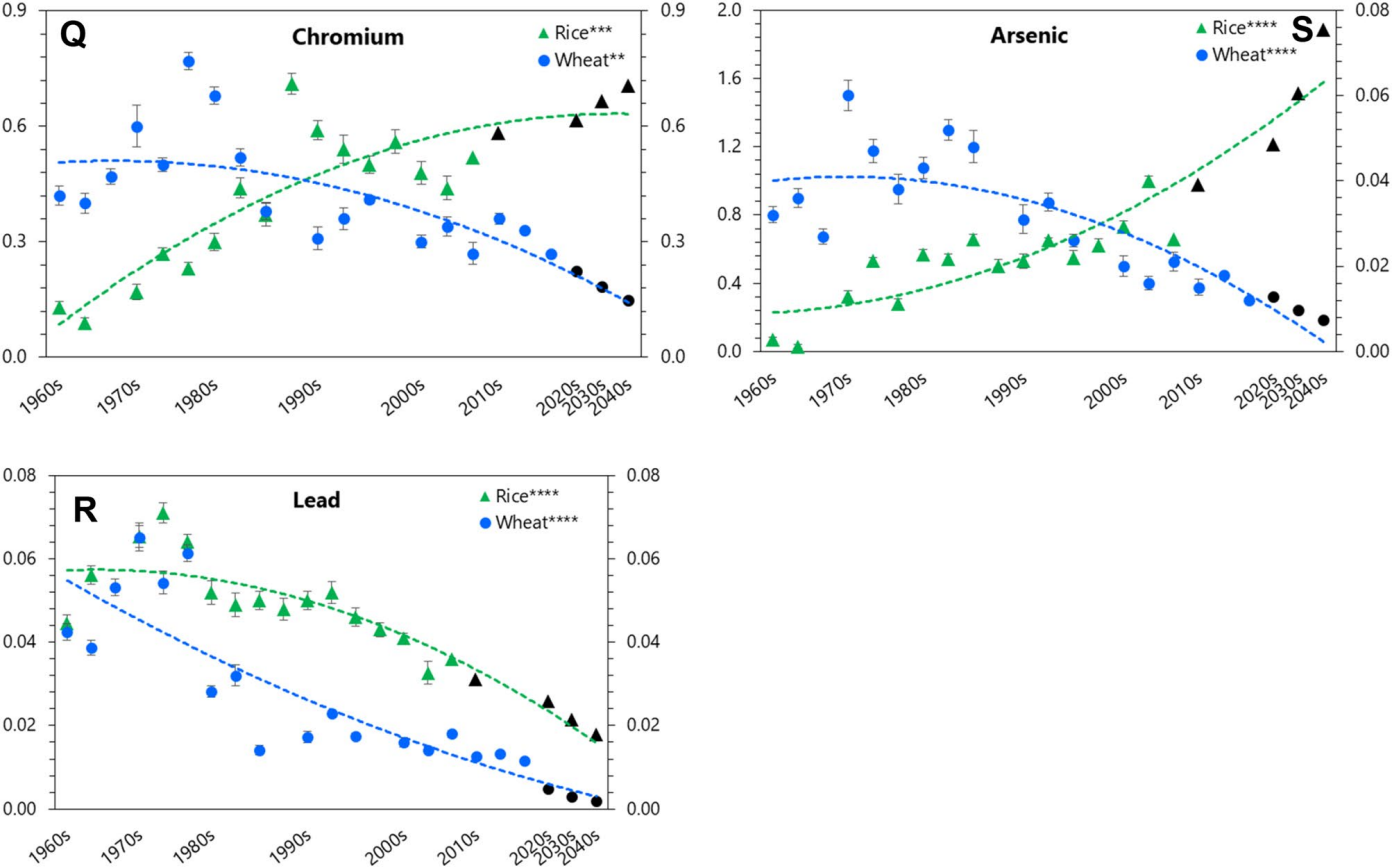
Ionome of rice and wheat grain in their cultivars released along the succeeding decades. Left and right vertical axis represents mineral concentration in wheat and rice grain, respectively. Horizontal axis represents decades of cultivar release for rice and wheat. Black triangles and dots represent projected change in ionome of rice and wheat grain along succeeding decades up to 2040. All units are in mg kg^−1^. Data are mean (*n* = 6). Vertical bars indicate standard error of mean. Asterisks indicate significant differences between the observed means of the decades for the cultivars of rice and wheat released up to 2000s and 2010s, respectively (*P < 0.05; **P < 0.01; ***P < 0.001; ****P < 0.0001).

Along decadal progression, there was a depletion for Ca, Zn, Fe, and Cu concentrations in both rice and wheat of newer-released cultivars than in their older ones (Fig. 1B,D,E, and F). In contrast, an augmentation for S concentrations was observed in newer-released cultivars in both rice and wheat (Fig. 1C). For example, Ca, Zn and Fe concentrations in grains of rice and wheat cultivars released in 1960s were 337.0, 19.9 and 33.6, and 492.3, 24.3 and 57.6 mg kg^−1^, which significantly decreased to 186.3 (45%-drop; *P* < 0.01), 13.4 (33%-drop; *P* < 0.001) and 23.5 (30%-drop; *P* < 0.0001), and 344.2 (30%-drop; *P* < 0.0001), 17.6 (27%-drop; *P* < 0.0001), and 46.4 (19%-drop; *P* < 0.0001) mg kg^−1^ in cultivars of 2000s and 2010s, respectively (Supplementary Table S1). On the other side, S concentration in grains of rice and wheat cultivars released in 1960s was 472.7 and 518.3 mg kg^−1^, which significantly increased to 653.6 (38%-rise; *P* < 0.001) and 1744.3 (236%-rise; *P* < 0.0001) mg kg^−1^ in cultivars of 2000s and 2010s, respectively.

#### Beneficial elements

With time, except Li and V concentration in rice, the concentration of beneficial elements depleted in newer-released cultivars over their older counterparts in grains of both rice and wheat (Fig. 1H–K). For example, Si concentration in grains of rice and wheat cultivars released in 1960s was 521.9 and 456.6 mg kg^−1^, which significantly depleted to 304.7 (42%-drop; *P* < 0.001) and 251.4 (45%-drop; *P* < 0.0001) mg kg^−1^ in cultivars of 2000s and 2010s, respectively (Supplementary Table S1). Except for Ga concentration in wheat grain, there was an upward trend for Ag and Ga concentrations in grains of newer-released rice and wheat cultivars (Fig. 1L–M).

#### Toxic elements

Along decadal progression, there was an upward trend for toxic elements concentrations, except Pb, in newer-released rice cultivars than in older ones; however, a reverse downward trend for Pb, Cr, and As concentration was observed in grains of newer-released wheat cultivars (Fig. 1N–S). For example, As concentration for rice cultivars released in 1960s was 0.05 mg kg^−1^, which significantly increased to 0.80 (1493%-rise; *P* < 0.0001) mg kg^-1^ in cultivars of 2000s; while, its concentration significantly decreased from 0.032 mg kg^−1^ in wheat cultivars released in 1960s to 0.015 mg kg^−1^ (53%-drop; *P* < 0.0001) in cultivars of 2010s (Fig. 1S; Supplementary Table S1). Aluminum (Al) concentration in grains of rice cultivars significantly increased from 29.3 mg kg^−1^ in 1960s to 52.1 (78%-rise; *P* < 0.0001) mg kg^−1^ in 2000s, and again, its concentration in grains significantly increased from 32.6 mg kg^−1^ in wheat cultivars released in 1960s to 44.6 mg kg^−1^ (37%-rise; *P* < 0.001) in cultivars of 2010s (Fig. 1O; Supplementary Table S1). In contrast, grain Pb concentration significantly depleted from 0.051 to 0.037 mg kg^−1^ (27%-drop; *P* < 0.0001) in rice and from 0.045 to 0.012 mg kg^−1^ (72%-drop; *P* < 0.0001) in wheat cultivars released in past more than 50 y (Fig. 1R; Supplementary Table S1). Pair-wise correlation between all the measured mineral elements showed positive and negative relations for both the cereals (Supplementary Tables S2–S3).

### Analyzing trend in grain yield and harvest index in landmark cultivars

**Figure 2:**
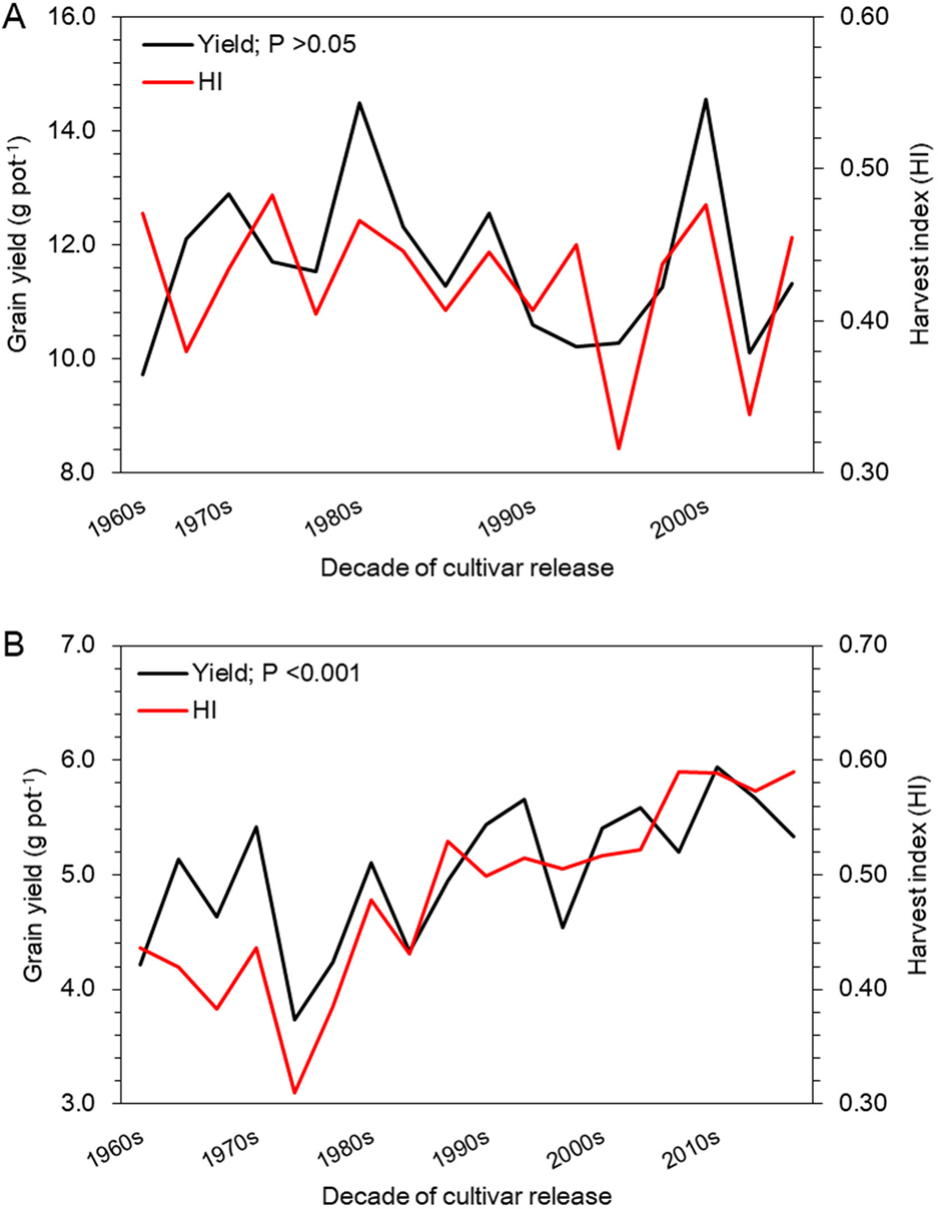
Yield and harvest index of the experimental rice and wheat cultivars at maturity. (**A**) Rice (**B**) Wheat. Data are means (*n* = 6). Significance refers to differences between the observed means of the decades for the cultivars of rice and wheat released up to 2000s and 2010s, respectively.

Our results did not show a significant (*P* > 0.05) enhancement in yield and harvest index (HI) in rice cultivars released over the succeeding decades since GR (Fig. 2). However, grain yield and HI of wheat cultivars increased steadily and significantly (*P* < 0.001) in past more than 50 y, which showed 19% yield enhancement.

### Dietary significance of grain mineral composition

Results showed a depleted average daily intake (ADI) of most of the essential elements (except for S and Mn) through consumption of both rice and wheat, along decadal progression (Table 1; Supplementary Table S4). It also showed that consumption of rice and wheat cultivars released in 1960s could meet 14.0, 11.2, and 33.7% and 20.4, 26.5, and 56.9% of the recommended dietary allowance (RDA) for Ca, Fe, and Zn, respectively, which significantly decreased to 5.4 (*P* < 0.01), 5.6 (*P* < 0.0001), and 15.7% (*P* < 0.0001) and 9.1 (*P* < 0.0001), 13.7 (*P* < 0.0001), and 25.5% (*P* < 0.0001) in rice and wheat cultivars released in 2000s and 2010s, respectively. Within the beneficial elements, along decadal progression, ADI of Si and Ni also decreased significantly in cultivars of both rice and wheat. Contrarily, there was an enhancement in ADI of toxic elements through consumption of rice cultivars released in succeeding decades. For example, ADI for As sharply increased from 0.024 (13.2% RDA) mg d^−1^ in cultivars of 1960s to 0.262 (145.4% RDA; *P* < 0.0001) mg d^−1^ in cultivars of 2000s.

**Table 1:** Average daily intake (ADI) of the elements by human beings through consumption of rice and wheat grain along succeeding decades and recommended daily allowance (RDA) for the elements. Data are mean (*n* = 6). Data including RDA are expressed in mg day^-1^.

Element	ADI considering bioavailability estimates*							RDA	%RDA						
	1960s	1970s	1980s	1990s	2000s	2010s	*P* value		1960s	1970s	1980s	1990s	2000s	2010s	*P* value
1. Rice															
P	727.5	545.0	433.7	368.8	403.5	–	NS^#^	1000^29^	72.8	54.5	43.4	36.9	40.4	–	*P* < 0.01
Ca	139.8	120.7	69.8	68.2	53.6	–	*P* < 0.01	1000^29^	14.0	12.1	7.0	6.8	5.4	–	*P* < 0.01
S	90.5	77.8	76.0	83.3	86.8	–	*P* < 0.001	181^30^^∆^	50.0	43.0	42.0	46.0	48.0	–	*P* < 0.0001
Fe	1.7	1.4	1.1	0.9	0.8	–	*P* < 0.0001	15^29^	11.2	9.6	7.3	6.0	5.6	–	*P* < 0.0001
Zn	3.7	2.7	2.2	1.9	1.7	–	*P* < 0.001	11^29^	33.7	24.2	19.7	17.7	15.7	–	*P* < 0.001
Cu	0.43	0.50	0.30	0.23	0.20	–	*P* < 0.01	2^29^	21.7	24.9	15.1	11.4	10.2	–	*P* < 0.01
Mn	0.13	0.09	0.10	0.11	0.14	–	*P* < 0.001	4^29^	3.4	2.2	2.5	2.8	3.5	–	*P* < 0.001
Si	11.1	8.2	5.9	4.6	4.5	–	*P* < 0.001	17^31^^†^	65.3	48.0	34.7	27.1	26.5	–	*P* < 0.001
Ni	0.060	0.039	0.027	0.018	0.013	–	*P* < 0.001	0.5^32^	12.0	7.8	5.4	3.7	2.7	–	*P* < 0.0001
Li	0.0122	0.0116	0.0118	0.0169	0.0134	–	*P* < 0.01	1^33^	1.22	1.16	1.18	1.69	1.34	–	*P* < 0.01
V	0.00002	0.00002	0.00012	0.00052	0.00060	–	*P* < 0.0001	1.2^34^^†^	0.002	0.002	0.010	0.044	0.050	–	*P* < 0.0001
Ag	0.004	0.003	0.013	0.039	0.053	–	*P* < 0.0001	0.05^35^^†^	7.9	6.8	26.6	77.2	105.5	–	*P* < 0.0001
Ba	0.0064	0.0101	0.0115	0.0131	0.0109	–	*P* < 0.001	12^36^^§^	0.053	0.084	0.096	0.109	0.091	–	*P* < 0.001
Sr	0.040	0.034	0.044	0.055	0.056	–	*P* < 0.0001	4^37^^§^	0.99	0.85	1.10	1.38	1.40	–	*P* < 0.0001
Cr	0.0012	0.0021	0.0039	0.0046	0.0035	–	*P* < 0.001	0.18^38^^†^	0.65	1.15	2.19	2.55	1.97	–	*P* < 0.001
As	0.024	0.155	0.219	0.204	0.262	–	*P* < 0.001	0.18^39^^§^	13.2	86.2	121.5	113.3	145.4	–	*P* < 0.0001
Pb	0.092	0.076	0.063	0.052	0.043	–	*P* < 0.0001	0.24^40^^§^	1.11	1.29	0.90	0.78	0.56	–	*P* < 0.0001
Al	15.6	18.9	17.4	21.5	19.2	–	*P* < 0.0001	36^39^^†^	43.2	52.4	48.3	59.7	53.4	–	*P* < 0.0001
2. Wheat															
P	994.5	935.1	807.5	772.6	677.4	554.9	*P* < 0.05		99.4	93.5	80.8	77.3	67.7	55.5	*P* < 0.05
Ca	204.3	220.5	85.0	89.0	70.2	91.3	*P* < 0.0001		20.4	22.1	8.5	8.9	7.0	9.1	*P* < 0.0001
S	99.3	100.5	122.3	143.9	189.3	213.5	*P* < 0.0001		54.8	55.5	67.6	79.5	104.6	118.0	*P* < 0.0001
Fe	4.0	3.5	3.0	2.4	2.1	2.1	*P* < 0.0001		26.5	23.3	19.7	15.8	14.0	13.7	*P* < 0.0001
Zn	6.3	4.9	4.2	3.9	3.3	2.8	*P* < 0.0001		56.9	44.3	38.5	35.4	30.2	25.5	*P* < 0.0001
Cu	0.62	0.63	0.46	0.33	0.30	0.26	*P* < 0.0001		31.0	31.6	22.9	16.3	15.1	13.0	*P* < 0.0001
Mn	0.33	0.28	0.23	0.25	0.25	0.26	*P* < 0.001		8.2	7.0	5.8	6.3	6.2	6.5	*P* < 0.001
Si	9.7	13.6	12.6	6.1	4.2	3.4	*P* < 0.0001		57.2	80.3	74.3	35.9	24.9	20.1	*P* < 0.0001
Ni	0.040	0.053	0.037	0.022	0.015	0.017	*P* < 0.0001		8.0	10.6	7.3	4.3	3.1	3.3	*P* < 0.0001
Li	0.071	0.065	0.035	0.033	0.022	0.010	*P* < 0.0001		7.1	6.5	3.5	3.3	2.2	1.0	*P* < 0.0001
V	0.0012	0.0014	0.0009	0.0004	0.0003	0.0002	*P* < 0.0001		0.102	0.118	0.072	0.036	0.024	0.016	*P* < 0.0001
Ag	0.028	0.030	0.034	0.036	0.043	0.028	*P* < 0.01		55.3	60.2	68.7	72.3	85.9	56.0	*P* < 0.01
Ba	0.056	0.061	0.051	0.074	0.078	0.075	*P* < 0.0001		0.467	0.509	0.421	0.615	0.654	0.623	*P* < 0.0001
Sr	0.093	0.103	0.084	0.093	0.122	0.093	*P* < 0.0001		2.32	2.58	2.10	2.33	3.05	2.33	*P* < 0.0001
Cr	0.0046	0.0058	0.0046	0.0028	0.0023	0.0022	*P* < 0.01		2.54	3.21	2.53	1.56	1.28	1.21	*P* < 0.01
As	0.015	0.020	0.018	0.011	0.006	0.005	*P* < 0.0001		8.3	11.1	10.2	5.9	3.5	2.5	*P* < 0.0001
Pb	0.0024	0.0028	0.0011	0.0007	0.0006	0.0004	*P* < 0.0001		0.99	1.16	0.45	0.31	0.25	0.18	*P* < 0.0001
Al	17.4	15.4	14.1	15.2	15.3	15.2	*P* < 0.001		48.2	42.9	39.2	42.1	42.5	42.2	*P* < 0.001

*Not available for Li, Ag, and Al.

^#^nonsignificant.

^∆^Based on RDA for methionine (combined with cysteine) for adults set at 1.1 g day^−1^.

^†^Adequate intake (AI) in adults in mg day^−1^.

^§^Tolerable intake (TI) in adults in mg day^−1^.

### Mineral diet quality index (M-DQI)

**Figure 3:**
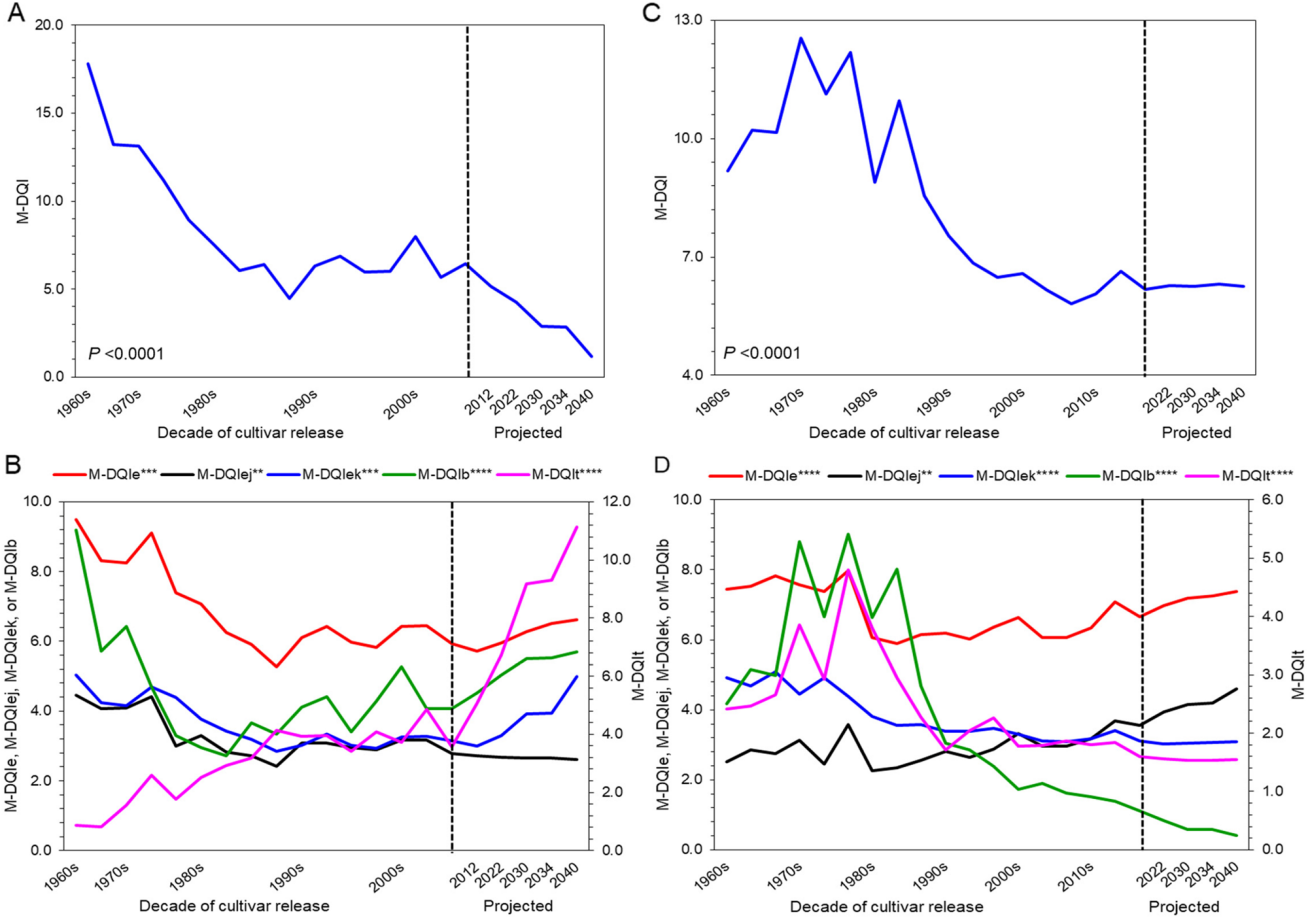
Mineral diet quality index (M-DQI) of rice and wheat grain of their cultivars released along succeeding decades. (**A**–**B**) Rice. (**C**–**D**) Wheat. M-DQI_e_, M-DQI_ej_, M-DQI_ek_, M-DQI_b_, and M-DQI_t_ represent M-DQI of essential, essential macro-elements, essential micro-elements, beneficial and toxic elements, respectively. Horizontal lines beyond the dashed vertical line extended up to 2040 represent projected M-DQI for rice and wheat cultivars released up to 2040. Data are mean (*n* = 6). Asterisks indicate significant differences between the observed means of the decades for the cultivars of rice and wheat released up to 2000s and 2010s, respectively (*P < 0.05; **P < 0.01; ***P < 0.001; ****P < 0.0001).

Results showed a depleting trend in M-DQI along succeeding decades for both the cereals (Fig. 3A and C). The M-DQI of the rice and wheat cultivars was 15.5 and 9.9 in 1960s, which significantly (*P* < 0.0001) dropped down to 6.7 (57%-drop) and 6.3 (36%-drop) in cultivars of 2000s and 2010s, respectively (Supplementary Tables S5–6). The M-DQI for essential (M-DQI_*e*_) and beneficial (M-DQI_*b*_) elements also showed a depleting trend along succeeding decades in both the cereals (Fig. 3B–D; Supplementary Table S14). Such depletion was sharp in rice than in wheat, and with micro-elements than macro-elements. For example, M-DQI_*e*_ of the rice and wheat cultivars was 8.9 and 7.6 in 1960s, which significantly (*P* < 0.0001) depleted to 6.3 (30%-drop), and 6.7 (12%-drop) in the cultivars of 2000s and 2010s, respectively (Supplementary Tables S5–6). Results also showed an augmentation of the M-DQI of toxic (M-DQI_*t*_) elements in rice (386%), but a depletion in wheat (30%) along the succeeding decades (Supplementary Table S6).

### Implications of M-DQI on human health

**Figure 4:**
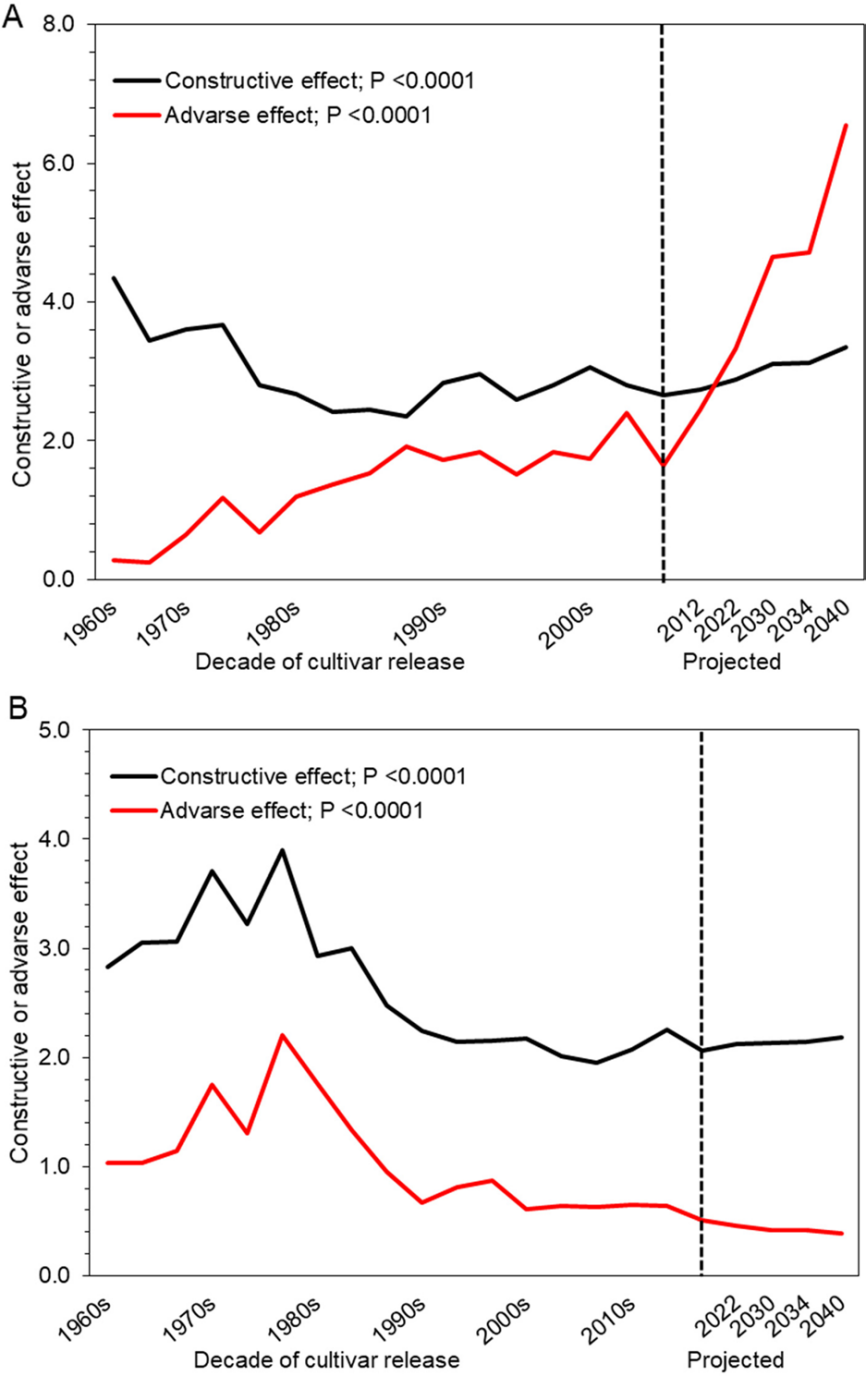
Manifestation of mineral diet quality index (M-DQI) on human health. (**A**) Rice. (**B**) Wheat. Horizontal lines beyond the dashed vertical line extended up to 2040 represent projected effects of M-DQI for rice and wheat cultivars released up to 2040. Constructive and adverse effect represents an average diet quality indexing scores of elements having beneficial and harmful effects on human health, respectively. Significance refers to differences between the observed means of the decades for the cultivars of rice and wheat released up to 2000s and 2010s, respectively.

We also quantified the constructive and adverse effects of M-DQI on human health, and observed a downward trend in both the effects for wheat over the past 50 y (Fig. 4). However, for rice, a downward trend in constructive effect, but an upward trend in adverse effect was observed along the succeeding decades (Fig. 4). The upward trend was sharp for the adverse effect than the downward trend for the constructive one in rice; while in wheat, the downward trend was sharp for the constructive than for the adverse effect of M-DQI on human health (Supplementary Table S6). For example, in rice, the constructive effect of M-DQI fell (*P* < 0.0001) from 3.90 in cultivars of 1960s to 2.84 in the cultivars of 2000s; while, the adverse effect enhanced (*P* < 0.0001) from 0.26 in 1960s to 1.93 in 2000s (Supplementary Table S5).

## Discussion

### Changes in grain nutrient concentration in landmark cultivars over time of release

Evaluation of grain nutrient concentration helps to decipher enhancement of goodness or otherwise in elemental composition brought out by physiological aberrations and/or genetic modifications of a crop cultivar. We observed a significant depletion in the concentration of essential (P, Ca, Fe, Zn, and Cu) and beneficial (Ni and Si) elements, but an accretion in toxic ones (As, Cr, Ba, Sr, and Al) in rice cultivars with time since 1960s; while in wheat, there was a depletion in most (excepting Ba and Sr) of the mineral elements analyzed. Similar depletion in nutrient concentration of cereal cultivars, over the past 50 years or so, was also documented by others^20,25,26,41,42^. Most of them reasoned it a ‘dilution effect’ caused by an enhancement of yield. However, we noticed no significant enhancement in yield and HI in rice, but in wheat cultivars released over the period. Thus, at least for rice, changes in grain nutrient concentration over time cannot be substantiated by the notional ‘dilution effect’. Again, our experimental soil contained adequate amount of plant available nutrients (Supplementary Table S7) and therefore, ability of the tested cultivars to sequester those nutrients was not constrained by their supply in soil, but perhaps related to incapacity of the cultivars’. Earlier a few researchers^18,25^ reported a similar depletion in essential grain minerals along time in wheat and rice coincided with the introduction of input-responsive, short-statured, high-yielding cultivars, and not with their inadequacy in soil supply. It essentially emphasizes that selection for yield and stress tolerance traits for many decades may not bring positive association always with grain mineral contents and subsequently requires a concurrent selection in cereal breeding process. Recent reports^12–14,43–45^ also suggest concurrent selection of both high yield and high grain mineral density (Zn, Fe in particular) in modern cereal breeding program may curb mineral inadequacies in humans.

During the green revolution period, insertion of a gibberellin-insensitive dwarfing-gene in rice (*sd 1* gene)^46^ and wheat (*Rht* gene)^47^ led to a higher distribution of photosynthates in to the grains thereby increasing the HI. However, it is implausible to have a pleiotropic effect of those dwarfing-genes on the uptake of nutrients by the genetically improved cultivars. On the other side, recent reports confirmed an impairment of screening capacity for the toxic elements at roots^2,48^ and regulatory mechanisms for their transport from shoot to grains in high-yielding cereal cultivars, rice in particular^49^. Possibly, it occurred due to a loss of natural evolutionary defense mechanisms of the landmark cultivars against the toxicants amid continuous genetic tampering under the modern breeding program^28^. Our observed depletion in the concentration of essential vis-à-vis accretion of toxic elements in grains of the cereal cultivars released over time may be a fall out of the above phenomena. Furthermore, a contrasting trend in grain toxic element concentration between the two staples may also be due to different toxic element uptake mechanism since rice was grown anaerobically while wheat aerobically. Anaerobic condition often enhances phyto-availability of toxic elements in soils^50^ and their subsequent uptake by plants^2^.

Previous reports^51,52^ on ionomics confirmed that elements with similar biological relevance and chemical properties (i.e., chemical analogs) share similar plant uptake mechanisms, transporters and thus, remain highly correlated. However, to our surprise, the pair-wise correlation between chemical analogs (e.g., Ca/Sr, Ca/Ba, Sr/Ba, Cu/Ag, Al/Ga, Si/Pb, and P/As) as well as between biologically associated elements (e.g., P/Ca, P/V, Ca/S, Ca/Si, Fe/S, Fe/Mn, and Fe/V, for their co-involvement in particular physiological or biochemical functions)^53–56^ appeared to be weak and even negative, for both the cereals (Supplementary Tables S2, S3, and S8). In addition, the correlation between Ca and Mn (chemical non-analog), which share intracellular transporter for their supply into developing grains^57^, was also weak and negative for both rice and wheat. This inverse or weak relation was more prevalent in rice (r = − 0.39; *P* > 0.05) than wheat (r = − 0.10; *P* > 0.05). These disjointed relations among the chemical analogs and biological associates are perhaps indicating an impairment in the genetic regulation of the grain mineral accumulation, silently and inadvertently caused in course of the past breeding programs.

To substantiate our claim further, we plotted soil concentration against transfer coefficient (TC) of those mineral elements and found, in majority, weak and inverse relations among them (Supplementary Fig. S1). It reiterated that the depleting trend in the grain nutrient concentration was caused by the plant rather than the soil factors. This is mainly because modern plant breeding has historically always been biased towards enhancing agronomic yield and disease-pest resistance^7^ and traits related to the grain mineral quality has largely been overlooked^7,25^. Evidences of erosion of the genetic basis for grain mineral accumulation in modern-bred cereals are not rare^21,22,58^. This breeding dilemma have recently been addressed, to some extent, by biofortifying staples with essential nutrients such as Zn-enriched rice^44,59,60^, wheat^43,61,62^, maize^63^, Fe-enriched rice^64,65^, and both Fe- and Zn-enriched rice and wheat^14^ at the national and international levels. However, future research should be focused on consumer acceptance of these biofortified crops following dissemination and their wide-scale adoption by the farming community.

We tried to assess the impact of such upward or downward trend of those mineral elements concentrations in years to come using a machine learning tool (i.e., Grey modelling). For example, if the observed decadal drop for Ca, Zn, and Fe concentration continues, it would result an additional drop of 57.0, 23.0, and 40.0% in cultivars of rice, and 22.0, 13.0 and 24.0% in cultivars of wheat by next two decades (i.e., 2040), respectively (Supplementary Table S9). On the other side, concentration of As, Cr, Sr, and Ba is projected to be increased by 135.0, 47.0, 160.0, and 63.0% in rice; while in wheat, concentration of Ba, Sr, and Al is projected to be increased by 67.0, 46.0, and 15.0%, respectively. Our projections thus indicate worsening of grain nutritional quality for both the cereals, perhaps making them impoverished for human consumption in near future.

### Dietary significance of the changes in grain nutrient concentration on human health

The altered grain nutrient concentration (ionome) eventually left a serious effect on the dietary significance of the two staples causing a depletion in average daily intake (ADI) of essential and beneficial elements. Such depletion was higher with beneficial (~ 68.0 and 73.0% in rice and wheat, respectively) than essential (~ 44.0 and 47.0%) elements, and with macro-elements (~ 53.0 and 50.0%) than micro-elements (~ 51.0 and 45.0%) (Supplementary Table S10). If this depletion in ADI continues unabated, it would further deplete 3.0, 4.0, and 2.0% and 1.7, 2.0, and 3.0% RDA of Ca, Zn, and Fe in cultivars of rice and wheat by 2040, respectively (Supplementary Table S4). It is noteworthy to indicate that the observed depletion in ADI is the result of the depleting grain mineral density of the cereals and falling per capita cereal consumption by the Indians^66^ (Supplementary Table S14) over the past 50 y. Further, over the years, a similar trend of lesser consumption of nutrient-rich cereals (sorghum and other millets)^4,11,67^ could altogether put the Indian population at higher risk of nutritional insecurity.

In contrast, there was an accretion in ADI of toxic elements, and it was higher with rice than with wheat in the past more than 50 y (Supplementary Table S10). If it continues as such, it would result 354.0, 23.0, and 2.0% additional accretion compared to the tolerable intake (TI) of As, Al, and Sr in cultivars of rice by 2040, respectively (Supplementary Table S4). Of the two cereals, wheat thus suffered the most with higher depletion for ADI of essential and beneficial elements; while, rice impoverished with a higher accretion for ADI of the toxic ones.

How do these results impact human health? To unearth the dietary consequences of the depleted concentration of essential and beneficial vis-à-vis accrued concentration of toxic minerals in the modern-bred cultivars of rice and wheat, a mechanistic model-based mineral diet quality index (M-DQI) and its possible impacts on human health was evaluated. Along the succeeding decades, a higher depletion in M-DQI for rice was mainly due to a higher accretion of toxic elements by rice over wheat cultivars. If this trend continues unhindered, it would result an additional depletion of ~ 77.0% M-DQI in cultivars of rice (Supplementary Table S6). In contrast, our model projects no further depletion in M-DQI for wheat cultivars.

We also tried to assess the positive (constructive) and negative (adverse) impacts of M-DQI separately on human health. Our model showed that rice consumption could pose higher adverse effects over the constructive effects on human health and well-being, mainly due to ingestion of more toxicants (Supplementary Table S5). Of late, it assumes significance for widespread contamination of precious soil resources with toxic elements, and, in turn, food grown on to it due to increasing industrialization and urbanization at an alarming rate in India^68^. There is strong evidence that oral ingestion of metal toxicants (As, Cr, Ba, and Sr) imposes toxic effects like lung cancers or chronic respiratory diseases^69^, cardiovascular diseases^70,71^, hyperkeratosis^72^, renal toxicity^73^ and impaired bone calcification^74^. As a fallout of the above phenomena, a natural high reliance on rice-diet could put the Indian population at higher risks of having non-communicable diseases (NCDs) like dermal, respiratory, renal, cardiovascular, or musculoskeletal ones (Supplementary Fig. S2A), which would worsen the country’s already high disease burden.

On the other hand, an intense downward trend in constructive effect of M-DQI up to 2000s in wheat cultivars reveals its declining nutritional benefits on human health due to depleted dietary intake of essential and beneficial elements in conjunction with its decreased consumption (Supplementary Table S5). However, its slight improvement was noticed in wheat cultivars released in 2010s, which is projected to remain stable till 2040. It is well known that P, Ca, Si, and V play important role for bone formation^75–78^, Zn for immunity, reproductive and neurological development^5,79^, and Fe for hemoglobin formation^80^. Therefore, depleted constructive effect of M-DQI could result higher prevalence of NCDs related to neurological, reproductive, iron deficiency anemia, and musculoskeletal system among the Indians relying on wheat-diet (Supplementary Figs. S2B–S3B). Our claim for higher risks of NCDs corroborates with a report published by the Indian Council of Medical Research (ICMR), Government of India^81^ indicating ~ 25% rise in it from 1990 to 2016 among the Indian population. It assumes a grieving concern considering the fact that rice–wheat together provides > 50% of daily energy requirements for Indian population. However, a simultaneous downward trend in adverse effect of M-DQI along succeeding decades, including projection, indicates a lower exposure for the Indian population to metal toxicants through wheat-diet (Supplementary Fig. S2B). Certainly, this may perhaps not be sufficient to significantly offset the depleted constructive effect of M-DQI for the Indian population.

## Conclusions

These results suggest that modern breeding agenda for genetic gains in the yield of rice and wheat in India have tended to reduce concentration of essential and beneficial elements, but to enhance toxic elements concentration in grains. Consequently, it dented the dietary value of the major staples (i.e., rice and wheat) to the Indian population in the past 50 years or so. Our study further highlights worsening of grain mineral diet quality for both rice and wheat up to 2040. This necessitates urgency of the efforts to improve the grain mineral density, at least, essential nutrients, of these staples through genetic interventions for sustenance of nutrition and human health in the years to come. The favourable alleles that were ‘left behind’ during the modern breeding process may be bred back into the cultivars, and grain nutrient profile should mandatorily be evaluated before release of a cultivar in near future.

## Materials and methods

### Experimental site, climate, and soil characteristics

An outdoor experiment was conducted in clay pots, having 7.0 kg capacity, at the University Research Farm (22°6′ N, 88°3′ E, 9.7 m amsl), West Bengal, India. The site experiences hot and humid climate with annual average rainfall of ~ 1500 mm, and maximum and minimum monthly temperatures of 36.0 °C and 12.0 °C, respectively. The soil used in this experiment was collected from top layer (0–20 cm) of a university land that had remained fallow for the past 2 years, without history of fertilization. It was classified as *Aeric Endoaquept* according to Soil Taxonomy (United States Department of Agriculture), and loamy in texture, and had pH 6.2, soil organic C 4.2 g kg^−1^, and phyto-available N 68.0 mg kg^−1^, P 11.0 mg kg^−1^, K 120.0 mg kg^−1^, Zn 3.0 mg kg^−1^, Fe 61.0 mg kg^−1^, Cu 3.8 mg kg^−1^, and Mn 6.7 mg kg^−1^ (Supplementary Table S11). Prior to the experimentation, the collected soil was air dried, pulverized and homogenized by repeated mixing and 5.0 kg of soil was filled in clay pots with black polyethylene sheeting.

### Cultivars and crop husbandry

The landmark high-yielding cultivars of rice (*Oryza sativa* L.) and wheat (*Triticum aestivum* L.) grown each on at least 5.0 mha of land in various states of India in successive decades of the past more than 50 y since GR were used (2–4 cultivars in each decade) for the experiment (Supplementary Fig. S4). The seeds for the landmark high-yielding cultivars of rice (*n* = 16) and wheat (*n* = 18) were acquired from the Gene-bank maintained by the Indian Council of Agricultural Research (ICAR)-National Rice Research Institute (NRRI), Cuttack, Odisha and ICAR-Indian Institute of Wheat and Barley Research (IIWBR), Karnal, Haryana, respectively. Major characteristics of the landmark cultivars with their parentage and year of release are presented in Supplementary Table S12.

To grow rice, seedlings were raised in small pots with 2.0 kg soil separately for all the 16 cultivars, which showed > 97% germination for both the season. Watering was done, as and when required, using water of a deep-tube well. One spray of 2% urea solution was given at 15 days old seedlings. Twenty-three days old seedlings were transplanted in a hill of three plants per pot, containing 5.0 kg soil, with three replications in the *kharif* (rainy) season of 2018 and 2019. Pot experiment was preferred to nullify differences in nutrient acquisition by crop-plants influenced by fertility gradient and other variables, which is, otherwise, experienced in field experiments. Fertilizers (N, P, and K at 80.0, 17.5 and 33.4 kg ha^−1^) were applied as per the recommended practice in all the pots on soil test basis. Nitrogen (N) was applied in two equal splits: half-dose at the time of transplanting, and the other-half at maximum tillering stage (21–23 days after transplanting, DAT), through diammonium phosphate (DAP; 18.0% N) and urea (46.0% N). The entire amount of P and K [as DAP (20.1% P) and muriate of potash (50.0% K), respectively] was applied at the time of transplanting. Irrigation was given, as and when required, using water from the said deep-tube well maintaining a water level of ~ 5.0 cm height in the pots from transplanting to grain filling stage. Manual weeding was preferred to control the weeds. Diseases and pests were not noticed in both the growing seasons.

After harvesting aboveground biomass of rice, roots were removed and the soil from each pot was again pulverized by breaking the hard clods and left in the respective pots without black polyethylene sheet for growing wheat, with all the same three replications in the *rabi* (winter) of 2018 and 2019. For wheat growing, seeding (five seeds per pot) was done with the help of a hand dibbler up to 1.0 cm depth, which showed > 93% germination for both the season, and later seedling population were thinned to only three plants per pot. Two additional set of pots used for wheat cropping was also managed similarly as in rice cropping, by growing a present-day popular rice cultivar ‘*Shatabdi*’ recommended for West Bengal condition. Fertilizers (N, P, and K at 120.0, 26.2 and 50.0 kg ha^−1^) were applied on soil test basis as per the recommended practice in all the pots. One-half of the N and the entire amounts of P and K were applied at the time of seeding and the other half of N at tillering stage (23–25 days after sowing, DAS), using the same sources of fertilizers as applied in rice. Irrigations were given, as and when required, to the pots, using water from the same source used in rice from seeding to grain filling stage. Weeding was done by hands and no diseases and pests were noticed in both the growing seasons.

### Grain sampling and digestion

At physiological maturity, all plants of the tested cereals per pot were harvested at 5 cm height from base with a pair of stainless steel scissors keeping panicles (rice)/heads (wheat) intact, separated in to shoots and grains manually, and weighed for their yield and placed in labelled clean paper bags. The harvested grains were thoroughly cleaned by winnowing through hot air, and subsequently stored in newly labelled clean brown paper bags. Fresh grains stored in paper bags were sundried for 5 days to achieve a moisture content < 14%.

The rice grains were subjected to hulling and milling to get polished rice. The hulling and milling operations were performed using a compact rice mill (THU-35C, Satake, Japan) running for 30 s for each step to obtain polished rice. The equipment was cleaned after each sample using a clean brush and/or muslin cloth to avoid cross contamination^82^. The polished rice thus prepared and the whole wheat grains of all the tested cultivars were washed firstly with dilute acid (0.1 M HCl; Merck, GmbH, Germany) and secondly with Type-1 (ultrapure) water (18.2 mΩ cm^-1^), and placed in labelled clean brown paper bags and dried to constant weight in an oven at 65 °C. After drying, samples (3 g) were ground in a stainless steel grinder by maintaining its cleanliness, placed in labelled clean brown paper bags and stored in a dry place till further analysis. The representative ground samples (0.5 g) were dry-ashed in porcelain crucibles (in a muffle furnace at 550 °C for 5 h) and dissolved in 6.0 M HCl (Merck, GmbH, Germany) solution^83^. Following ashing, the crucibles were washed thrice with Type-1 water and filtered through Whatman No. 42 filter paper, and the filtrate was diluted to 50 mL (1:100 w/v) with Type-1 water, and stored in polypropylene bottles till elemental analysis. Prior to use, porcelain crucibles, glassware and polypropylene bottles were properly acid-washed (1.0 M HCl; Merck, GmbH, Germany) to ensure their cleanliness and to avoid any contamination^82^.

### Soil sampling and extraction

Soil samples were collected from each of the three replicated pots after harvesting of wheat grown in *rabi* season of 2019. Soils were analyzed for phyto-available 19 elements, as for grain samples, by extracting with Mehlich-3 multi-element extractant solution (0.2 N HOAc, 0.25 N NH_4_NO_3_, 0.015 N NH_4_F, 0.013 N HNO_3_, and 0.001 M ethylenediamine tetraacetic acid) adjusted to pH 2.5^84^. For extraction purpose, a measured quantity of soil was shaken in a horizontal shaker (200 reciprocation min^−1^) for 5 min with soil to extractant ratio 1:10 (w/v) at 27 °C in polypropylene tubes, then centrifuged at 1200 rpm (R-8C, Remi, India) and filtered through Whatman No. 42 filter paper, and stored in polypropylene bottles till analysis. Glassware and polypropylene tubes/bottles, prior to their use, were properly acid-washed (1.0 M HCl, Merck, GmbH, Germany) to ensure cleanliness and to avoid cross contamination from previous stored samples^82^. Phyto-available element concentrations in the soils are given in Supplementary Table S7.

### Multi-element analysis

Extracts of the polished rice, wheat grain, and soils were analysed for concentrations of 19 elements viz., Ag, Al, As, Ba, Ca, Cr, Cu, Fe, Ga, Li, Mn, Ni, P, Pb, S, Si, Sr, V, and Zn by an ICP-OES (Optima DV-2100, Perkin Elmer, USA). The elements were categorized in text as essential macro-elements (P, Ca, and S), essential micro-elements (Fe, Zn, Cu, and Mn), beneficial elements (Li, Si, Ni, and V), ultra-beneficial elements (Ag and Ga), and toxic elements (Ba, Sr, Cr, Pb, As, and Al), in relation to human nutrition^50,85–88^.

The ICP-OES is featured with simultaneous background correction, inter-element correction, multi-component spectral fitting and automated rinse function that significantly enhances analytical performance and minimizes potential interferences or cross contamination. For quality control during analysis, a reference soil material (ERM-CC 141) from the Institute for Reference Materials and Measurements, European Commission, Belgium was used, and the samples were analysed in triplicates and the recoveries were recorded. The recoveries varied from 95 to 98% for all the tested samples. External multi-element calibration standard (*Trace*CERT®, Merck, GmbH, Germany) included (33 elements) Ag, Al, As, B, Ba, Be, Bi, Cd, Ca, Co, Cr, Cs, Cu, Fe, Ga, In, K, Li, Mg, Mn, Na, Ni, P, Pb, Rb, S, Se, Si, Sr, Te, Tl, V, and Zn, at a concentration of 10.0 mg L^−1^. For instrumental precision, standards were reintroduced after each 10-run of samples. Preparation blank and Type-1 water blank was used during sample run to detect any contamination. Grain concentrations of elements K, Mg, Na, and B exceeded the upper limit of standard (i.e., 10.0 mg L^−1^) in the diluted (1:100 w/v) samples and thus excluded from the working element dataset, to avoid discrepancy in measurement of element concentration arising due to additional dilution. Again, due to lack of desired sensitivity of the instrument, concentrations of elements Bi, Cs, In, and Te were excluded from the working element dataset. Further, grain concentrations of elements Be, Cd, Co, Rb, Se, and Tl were below their limits of detection (LOD) (Supplementary Table S13) and thus excluded from the working element dataset.

### Average daily intake (ADI)

The average daily intake of elements via polished rice and/or whole wheat consumption was calculated using a general equation commonly used by the nutritionists:

1 $${\text{ADI}}\left( {{\text{mg day}}^{{ - {1}}} } \right) = {\text{IR}} \times {\text{C}}_{e} \times {\text{B}}_{f}$$

IR is the cereal intake rate for an Indian adult obtained through household consumption data from successive rounds of the National Sample Survey, Ministry of Statistics and Programme Implementation, Government of India in the past 50 y since the GR and expressed in kg capita^−1^ day^−1^ (Supplementary Table S14). The projected household cereal consumption data is obtained from the National Institutions for Transforming India (NITI) Aayog, Government of India^66^. C_*e*_ is the concentration of elements (mg kg^−1^) in the polished rice or whole wheat grain samples. B_*f*_ is the bioavailability factor of the elements to human (Supplementary Table S15) and its consideration is exclusively based on the assumption that all cultivars have the same level of bioavailability, irrespective of the mineral elements considered.

### Mineral diet quality index (M-DQI)

The mineral diet quality index was computed based on the concentration of mineral elements in the grains of the cereals. In this frame, the goal was to integrate the grain concentrations of various minerals, comprised of essential (P, Ca, S, Zn, Fe, Cu, and Mn), beneficial (Ni, Si, Li, and V), and toxic (Al, As, Ba, Cr, Pb, and Sr) elements, in to a unit-less number indicating an improvement or impoverishment in mineral diet quality of the cereals in the past more than 50 y or so.

Firstly, in our proposed model for the index construction, within each selected mineral element, the observed grain concentration was divided by a so-called ‘average literature concentration (C_*a*_)’ of the element under consideration. Over 500 research articles were rigorously surveyed on the popular digital libraries like Google, Science Direct, Springer, PubMed, Researchgate, and Academia for identification of their range and average literature concentration in rice and wheat grains (Supplementary Table S16). The outliner concentrations reported in some studies were not considered in computing the C_*a*_. In this model, Ag and Ga were excluded due to unavailability of their C_*a*_.

Secondly, the unit-less score so generated was assigned with a weight factor (0–1) to each mineral contributing to the diet quality. A coefficient so generated was termed as diet coefficient (Q). The Q value of a mineral element is fixed based on three main assumptions: (i) the scientific evidence demonstrating a specific health benefit associated to the intake of that particular mineral nutrient (i.e., high for essential elements, intermediate for beneficial elements, and low for toxic elements); (ii) the contribution that the experimental cereals gives for the daily intake of that particular mineral nutrient; and (iii) the bioavailability of particular mineral nutrient through plant based-diet. Weight factors were assigned separately for each of the three assumptions as per the authors’ experience and subsequently added together to get Q value for a particular mineral element (Supplementary Table S17). The value of Q can be eventually adapted for different populations in consideration of their average dietary intake of cereal in the overall diet.

Summarizing, the mineral diet quality index for essential, beneficial, and toxic elements is given by our own proposed equations:

2 $${\text{M-DQI}}_{e} = \sum {\left[ {\left( {{\text{C}}_{ej} /{\text{C}}_{a} } \right) \times {\text{Q}}_{j} } \right]} + \sum {\left[ {\left( {{\text{C}}_{ek} /{\text{C}}_{a} } \right) \times {\text{Q}}_{k} } \right]}$$

3 $${\text{M-DQI}}_{b} = \sum {\left[ {\left( {{\text{C}}_{b} /{\text{C}}_{a} } \right) \times {\text{Q}}_{b} } \right]}$$

4 $${\text{M-DQI}}_{t} = \sum {\left[ {\left( {{\text{C}}_{t} /{\text{C}}_{a} } \right) \times {\text{Q}}_{t} } \right]}$$

where M-DQI_*e*_, M-DQI_*b*_ and M-DQI_*t*_ is M-DQI for essential, beneficial, and toxic elements, and C_*ej*_, C_*ek*_, C_*b*_, and C_*t*_ is the grain concentration of an essential macro-element (*j*), essential micro-element (*k*), beneficial element (*b*), and toxic element (*t*), respectively. Q_*j*_, Q_*k*_, Q_*b*_, and Q_*t*_ is the computed diet coefficient for essential macro-elements, micro-elements, beneficial, and toxic elements, respectively.

Finally, after scoring the elements, the mineral diet quality index is given by our own proposed an additive equation:

5 $${\text{M-DQI}} = \left[ {{\text{M-DQI}}_{e} + {\text{M-DQI}}_{b} } \right] - {\text{M-DQI}}_{t}$$

### Manifestation of M-DQI on human health

In this frame, the impacts, either positive or negative, of M-DQI on human health was quantified with subsequent steps. Firstly, the specific role(s) of the measured mineral elements on human health system was ascertained through intensive literature survey (Supplementary Table S18). Secondly, the role(s) was segregated into two groups: one with beneficial (i.e., constructive) effect and the other with harmful (i.e., adverse) effect. Thirdly, diet quality indexing scores of particular elements, having either beneficial or harmful effects, are summarized separately and a mean indexing value of these effects was computed to quantify the manifestation of M-DQI on a human health system through consumption of cereals in the past more than 50 y or so.

### Calculations used in the study

Harvest index (HI), a ratio of economic yield to biological yield, was calculated using a general equation commonly used by the agronomists:

6 $${\text{HI}} = {\text{grain yield}}/\left( {{\text{grain yield}} + {\text{straw yield}}} \right)$$

Transfer coefficient (TC) was computed to determine the relative translocation of an element from soil to grain of rice and wheat^2^.

7 $${\text{TC}}_{{\text{soil to grain}}} = {\text{nutrient concentration in grain}}/{\text{nutrient concentration in soil}}$$

### Statistical analysis

All data presented here are means of two growing seasons in each crop. One way analysis of variance (ANOVA) was conducted for the measured parameters having a set of quantitative dependent variables. Each dependent variable was differently analyzed by an independent factor namely decade and conducted the Tukey’s honest significance difference (HSD) test for comparing the means, using SPSS 16.0 (SPSS Inc., Chicago, USA) for Windows. Each measured parameters, comprising the decades, served as the source of variance. The hypothesis was to test that several means of the decades for the cultivars are equal at 5% level of significance. Simple correlation (Pearson) was worked out among the mineral concentrations in polished rice and whole wheat grain to elucidate their degree of relationship using SPSS 16.0.

We also made a projection for the mineral concentrations in rice and wheat grain and its impact up to 2040 with the rationales that there will be (i) no dramatic change in philosophical approach for modern cereal breeding, (ii) an increasing essential multi-element deficiencies in Indian soils due to exhaustion of nutrient reserves following GR, and (iii) an increasing soil contamination with heavy-metals due to industrialization and/or urbanization amid India’s ambition to become $40 trillion economy by 2047. To do the projection, we used Grey modelling since it works even with very few observations^89^, and the Grey model was implemented using R Package ‘Grey Model’^90^ in this study.

### Ethical approval

The seeds of rice and wheat cultivars were collected from the national Gene-banks as declared by the Indian Council Agricultural Research (ICAR) and their use in the present study complies with international, Indian, and/or institutional (herein BCKV, ICAR-NRRI, and ICAR-IIWBR) guidelines.

### Supplementary Information

Supplementary Information.

Supplementary Table S1.

Supplementary Table S4.

Supplementary Table S5.

Supplementary Table S12.

Supplementary Table S17.

## Data Availability

All of the data pertaining to the experiments are contained within the figures and supplementary materials.
