# Supplementary material for: Historical shifting in grain mineral density of landmark rice and wheat cultivars released over the past 50 years in India
Source: Sci Rep. 2023 Nov 30;13:21164. doi: 10.1038/s41598-023-48488-5 (PMC10689764; doi:10.1038/s41598-023-48488-5)
Supplement: Supplementary file 1 — Supplementary Information. [file 41598_2023_48488_MOESM1_ESM.pdf]

Supplementary Information (SI) for

Historical shifting in grain mineral density of landmark rice and wheat cultivars released over the past 50 years in India

Sovan Debnath, Ahana Dey, Rubina Khanam, Susmit Saha, Dibyendu Sarkar, Jayanta K. Saha, Mounissamy V. Coumar, Bhaskar C. Patra, Tufleuddin Biswas, Mrinmoy Ray, Madhari S. Radhika, Biswapati Mandal

This PDF file includes:

Supplementary Tables S2, S3, S6-S11, S13-16 and S18

Supplementary Figures S1 to S4

SI References

Supplementary Table S2. Pearson correlation coefficients (r-value) between 19 measured elements in rice

	P	Ca	S	Fe	Zn	Cu	Mn	Si	Ni	Li	V	Ag	Ga	Ba	Sr	As	Pb	Cr	Al
P	1.00																		
Ca	0.70**	1.00																	
S	-0.36	-0.44	1.00																
Fe	0.60**	0.69**	-0.69**	1.00															
Zn	0.75**	0.67**	-0.55*	-0.33	1.00														
Cu	0.51*	0.83**	-0.52*	0.78**	0.51*	1.00													
Mn	-0.05	-0.39	0.82**	-0.62*	-0.31	-0.59*	1.00												
Si	0.81**	0.82**	-0.61*	0.80**	0.77**	0.67**	-0.42	1.00											
Ni	0.72**	0.83**	-0.69**	0.81**	0.85**	0.67**	-0.47	0.94**	1.00										
Li	-0.54*	-0.55*	0.58*	-0.71**	-0.64**	-0.66**	0.39	-0.60*	-0.72**	1.00									
V	-0.43	-0.61*	0.90**	-0.76**	-0.63**	-0.71**	0.76**	-0.67**	-0.77**	0.80**	1.00								
Ag	-0.48	-0.64**	0.85**	-0.79**	-0.60*	-0.72**	0.77**	-0.68**	-0.76**	0.71**	0.90**	1.00							
Ga	-0.50*	-0.72**	0.81**	-0.83**	-0.73**	-0.74**	0.70**	-0.79**	-0.86**	0.82**	0.94**	0.86**	1.00						
Ba	-0.83**	-0.69**	0.54*	-0.79**	-0.88**	-0.59*	0.23	-0.80**	-0.87**	0.85**	0.70**	0.68**	0.77**	1.00					
Sr	-0.55*	-0.62*	0.89**	-0.87**	-0.70**	-0.72**	0.75**	-0.75**	-0.78**	0.73**	0.92**	0.89**	0.92**	0.72**	1.00				
As	-0.58*	-0.59*	0.74**	-0.79**	-0.84**	-0.46	0.64**	-0.76**	-0.79**	0.46	0.69**	0.70**	0.77**	0.70**	0.81**	1.00			
Pb	0.30	0.64*	-0.64**	0.67**	0.39	0.81**	-0.79**	0.46	0.47	-0.42	-0.67**	-0.77**	-0.64**	-0.34	-0.71**	-0.50*	1.00		
Cr	-0.85**	-0.72**	0.54*	-0.79**	-0.85**	-0.66**	0.27	-0.79**	-0.81**	0.80**	0.70**	0.66**	0.77	0.93**	0.74**	0.65**	- 0.49	1.00	
Al	-0.73**	-0.59*	0.74**	-0.71**	-0.77**	-0.54*	0.45	-0.78**	-0.83**	0.84**	0.83**	0.78**	0.81**	0.88**	0.79**	0.69**	- 0.43	0.82**	1.00

*P <0.05

**P <0.01

Supplementary Table S3. Pearson correlation coefficients (r-value) between 19 measured elements in wheat

	P	Ca	S	Fe	Zn	Cu	Mn	Si	Ni	Li	V	Ag	Ga	Ba	Sr	As	Pb	Cr	Al
P	1.00																		
Ca	0.37	1.00																	
S	-0.60*	-0.49*	1.00																
Fe	0.34	0.64**	-0.80**	1.00															
Zn	0.50*	0.55*	-0.80**	-0.17	1.00														
Cu	0.31	0.61**	-0.80**	0.79**	0.60**	1.00													
Mn	-0.50*	-0.10	0.75**	-0.47*	-0.55*	-0.53*	1.00												
Si	0.54*	0.38	-0.71**	0.58*	0.51*	0.70**	-0.77**	1.00											
Ni	0.46	0.69**	-0.67**	0.62**	0.57*	0.80**	-0.60*	0.87**	1.00										
Li	0.57*	0.67**	-0.89**	0.73**	0.81**	0.83**	-0.51*	0.61**	0.69**	1.00									
V	0.47	0.71**	-0.78**	0.76**	0.69**	0.84**	-0.57*	0.79**	0.90**	0.82**	1.00								
Ag	0.12	-0.57*	0.56*	-0.63**	-0.54*	-0.68**	0.27*	-0.40	-0.62**	-0.58*	-0.68**	1.00							
Ga	0.63**	0.36	-0.74**	0.64**	0.55*	0.65**	-0.80**	0.94**	0.80**	0.60**	0.81**	-0.36	1.00						
Ba	-0.28	-0.47*	0.85**	-0.79**	-0.72**	-0.75**	0.71**	-0.70**	-0.68**	-0.75**	-0.82**	0.70**	-0.72**	1.00					
Sr	-0.15	-0.33	0.77**	-0.73**	-0.68**	-0.63**	0.56*	-0.60**	-0.53*	-0.63**	-0.63**	0.74**	-0.62**	0.82**	1.00				
As	0.49*	0.37	-0.77**	0.50*	0.53*	0.71**	-0.76**	0.91**	0.77**	0.65**	0.74**	-0.42	0.85*	-0.74**	-0.62**	1.00			
Pb	0.47	0.79**	-0.78**	0.79**	0.70**	0.90**	-0.44	0.72**	0.87**	0.89**	0.91**	-0.66**	0.67**	-0.73**	-0.58*	0.66**	1.00		
Cr	0.49*	0.54*	-0.60**	0.57*	0.44	0.70**	-0.52*	0.84**	0.90**	0.64**	0.81**	-0.45	0.77**	-0.59**	-0.49*	0.65**	0.77**	1.00	
Al	-0.50*	-0.30	0.87**	-0.74**	-0.72**	-0.75**	0.81**	-0.70**	-0.71**	-0.74**	-0.81**	0.53*	-0.85**	0.82**	0.74**	-0.69**	-0.73**	-0.67**	1.00

*P <0.05

**P <0.01

Supplementary Table S6. Observed and projected change in mineral diet quality index (M-DQI) and its effects on human health system through consumption of rice and wheat cultivars released along succeeding decades

Attribute	Rice						Wheat					
	1960s	2000s	2040	Observed change (%) (1960-2000)	Projected change (%) (2010-2040)	Change in 80 years (%) (1960-2040)	1960s	2010s	2040	Observed change (%) (1960-2010)	Projected change (%) (2020-2040)	Change in 80 years (%) (1960-2040)
M-DQI	15.53	6.68	1.18	-57.0	-77.0	-92.0	9.86	6.29	6.25	-36.0	-0.30	-37.0
M-DQI$_e$	8.90	6.26	6.62	-30.0	+16.0	-26.0	7.60	6.70	7.39	-12.0	+5.90	-3.00
M-DQI$_b$	7.46	4.47	5.69	-40.0	+26.0	-24.0	4.77	1.34	0.41	-72.0	-50.0	-91.0
M-DQI$_t$	0.83	4.10	11.13	+386.0	+120.0	+1240.0	2.51	1.75	1.55	-30.0	-0.50	-38.0
Effect of M-DQI on human health												
Constructive	3.90	2.84	3.35	-27.0	+22.0	-14.0	2.98	2.13	2.19	-28.0	+3.00	-27.0
Adverse	0.26	1.93	6.55	+642.0	+167.0	+2420.0	1.07	0.60	0.38	-44.0	-17.0	-64.0

Supplementary Table S7. Concentration of Mehlich-3 extractable elements in soils sampled after harvest of wheat of 2019-20. Data are in mg kg^{-1}, summarized across all pots (n = 54).

Element	Range	Mean	Median	SD†	Variation§	CV¶	Adequate level in soil	Source
P	61.96-88.36	74.96	74.75	6.32	142.60	3.72	36.00-68.00	1
Ca	2071.20-2225.90	2142.16	2133.05	21.37	107.47	1.08	700.00-895.00	1
S	60.40-88.70	75.36	75.95	6.33	146.85	8.80	31.00-40.00	2
Fe	167.91-213.64	191.69	189.94	3.22	128.24	1.94	60.00-420.00	3
Zn	6.95-9.13	8.09	8.06	0.31	131.37	4.42	2.21-5.00	3
Mn	67.77-92.49	80.03	79.51	5.35	138.48	7.02	30.10-200.00	3
Cu	4.68-5.85	5.36	5.33	0.19	124.00	3.97	1.61-4.50	3
Li	0.01-0.07	0.039	0.04	0.009	700.00	25.71	-	
Si	233.30-277.90	256.53	257.25	10.37	119.11	4.28	14.00-207.00	4
Ni	1.10-1.55	1.36	1.38	0.09	140.90	7.2	<19.00	5
V	2.24-2.86	2.59	2.59	0.09	127.68	3.73	-	
Ag	0.01-0.05	0.027	0.030	0.008	500.00	30.40	-	
Ga	0.39-0.52	0.44	0.44	0.03	133.33	8.52	-	
Al	522.79-589.49	542.03	539.84	6.89	112.76	1.34	384.00-1825.00	6
Cr	0.28-0.41	0.35	0.35	0.02	146.43	5.48	<50.00	7
As	0.70-1.33	1.01	0.99	0.11	190.00	12.74	5.00-10.00	8
Pb	7.32-9.71	8.51	8.62	0.42	132.65	5.22	<85.00	9
Ba	30.96-35.58	32.44	31.92	0.58	114.92	2.57	<44.00	10
Sr	6.98-9.00	7.70	7.68	0.30	128.94	4.31	-	

†standard deviation

§[(maximum/minimum)] × 100

¶coefficient of variation

Supplementary Table S8. Pearson correlation coefficients (r-value) for grain composition of groups of mineral elements in rice and wheat

	Group II			Group XI		Group XIII		Group XIV		Group XV	
Rice	Ba	-0.69**		Cu	-0.72**	Al	0.81**	Si	0.46	P	-0.58*
	Sr	-0.62*	0.72**		Ag		Ga		Pb		As
		Ca	Ba								
Wheat	Ba	-0.47*		Cu	-0.68**	Al	-0.85**	Si	0.72**	P	0.49*
	Sr	-0.33	0.82**		Ag		Ga		Pb		As
		Ca	Ba								

*P <0.05

**P <0.01

Supplementary Table S9. Observed and projected change in grain mineral element concentrations of rice and wheat cultivars released along succeeding decades

Element	Rice			Wheat		
	Observed change (%) (1960-2000)	Projected change (%) (2010-2040)	Change in 80 years (%) (1960-2040)	Observed change (%) (1960-2010)	Projected change (%) (2020-2040)	Change in 80 years (%) (1960-2040)
P	-20	-19	-35	-13	-7	-19
Ca	-45	-57	-76	-30	-22	-45
S	+38	+29	+78	+236	+35	+353
Fe	-28	-40	-56	-19	-24	-39
Zn	-33	-23	-48	-30	-13	-39
Cu	-33	-68	-79	-35	-44	-63
Mn	+51	+157	+288	+23	+21	+48
Si	-42	-48	-70	-45	-60	-78
Ni	-68	-69	-90	-34	-68	-79
Li	+56	+95	+205	-77	-50	-88
V	+2600	+120	+5850	-74	-79	-94
Ag	+1490	+1530	+25785	+58	+53	+142
Ga	+300	+511	+2346	-57	-52	-80
Ba	+145	+63	+300	+108	+66	+246
Sr	+102	+160	+422	+57	+46	+130
Cr	+336	+47	+540	-26	-54	-65
Pb	-26	-52	-64	-73	-85	-96
As	+1500	+135	+3665	-53	-51	-77
Al	+78	+48	+163	+37	+15	+57

Supplementary Table S10. Net change in average daily intake (ADI) of the measured nutrient elements over the past 50 y in the cultivars of rice (1960–2000) and wheat (1960–2010).

Element	Rice		Wheat	
	ΔADI_{50} (mg day^{-1})*	% change	ΔADI_{50} (mg day^{-1})	% change
P	-323.97	-44.53	-439.55	-44.20
Ca	-86.22	-61.66	-113.01	-55.32
S	-3.70	-4.09	114.24	+115.10
Fe	-0.83	-49.66	-1.93	-48.45
Zn	-1.98	-53.39	-3.46	-55.23
Cu	-0.23	-53.11	-0.36	-57.95
Mn	0.01	+4.95	-0.07	-21.54
Si	-6.61	-59.51	-6.30	-64.81
Ni	-0.05	-77.80	-0.024	-58.52
Li	0.0011	+9.24	-0.061	-85.62
V	0.0006	+2714.90	-0.0010	-84.25
Ag	0.0488	+1232.18	0.0003	+1.19
Ba	0.0045	+70.32	0.019	+33.48
Sr	0.0164	+41.13	0.0004	+0.38
Cr	0.0024	+202.67	-0.0024	-52.44
As	0.2380	+1005.15	-0.0105	-69.73
Pb	-0.0013	-49.57	-0.0020	-82.18
Al	3.6516	+23.47	-2.19	-12.59

*ΔADI_{50} = ADI of an element in rice cultivars of 2000s or wheat cultivars of 2010s – ADI of that element in rice or wheat cultivars of 1960s.

Supplementary Table S11. Elemental composition of the soil prior to experimentation (*n* = 4)

Element	Unit	Value (*n* = 4)
Soil organic C	g kg^{-1}	4.2
Total organic C	g kg^{-1}	8.7
Total Si	g kg^{-1}	4.75
Total Al	g kg^{-1}	15.10
Total Ca	g kg^{-1}	6.12
Total S	g kg^{-1}	5.72
Total Fe	g kg^{-1}	20.31
Total P	mg kg^{-1}	663.11
Total Zn	mg kg^{-1}	44.32
Total Mn	mg kg^{-1}	398.54
Total Cu	mg kg^{-1}	39.20
Total Ni	mg kg^{-1}	62.06
Total Li	mg kg^{-1}	44.14
Total V	mg kg^{-1}	87.00
Total Co	mg kg^{-1}	40.10
Total Se	mg kg^{-1}	58.63
Total Ag	mg kg^{-1}	5.34
Total Ga	mg kg^{-1}	208.00
Total Pb	mg kg^{-1}	43.84
Total Cd	mg kg^{-1}	0.23
Total As	mg kg^{-1}	17.00
Total Cr	mg kg^{-1}	70.00
Total Ba	mg kg^{-1}	587.73
Total Sr	mg kg^{-1}	41.20
Total B	mg kg^{-1}	293.00

Supplementary Table S13. Element specific limits of detection (LOD) for concentrations of 29 elements measured in rice, wheat, and soil by ICP-OES

Elements	LOD (µg L^{-1})
K	2.00
Mg	1.00
Si	0.50
Al	2.00
Ca	2.00
S	5.00
Fe	0.50
P	5.00
Zn	1.00
Mn	0.10
Cu	1.00
Ni	1.00
Li	0.01
V	1.50
Co	0.40
Se	5.00
Ag	0.50
Ga	0.01
Pb	2.00
Cd	0.50
As	4.00
Cr	0.50
Ba	0.20
Sr	0.10
B	0.25
Na	1.00
Rb	2.00
Tl	5.00
Be	0.10

Supplementary Table S14. Pattern of mean cereal intake rate of an Indian adult in the past 50 y since the green revolution and its projection up to 2040

Decade	Year	NSS[#] round	Rural	Urban	National[$]	National mean for decade
			kg capita^{-1} day^{-1}			
1960s	1961-62	17th	0.585	0.416	0.554	0.532
	1964-65	19th	0.540	0.388	0.511	
1970s	1970-71	25th	0.512	0.379	0.485	0.463
	1972-73	27th	0.509	0.375	0.442	
	1973-74	28th	0.503	0.377	0.477	
	1977-78	32nd	0.508	0.387	0.448	
1980s	1983	38th	0.493	0.377	0.435	0.433
	1987-88	43rd	0.485	0.378	0.431	
1990s	1993-94	50th	0.447	0.353	0.400	0.390
	1999-2000	55th	0.423	0.335	0.379	
2000s	2000-01	56th	0.413	0.333	0.373	0.369
	2001-02	57th	0.407	0.327	0.367	
	2002(2)	58th	0.403	0.327	0.365	
	2003	59th	0.410	0.330	0.370	
	2004(1)	60th	0.413	0.333	0.373	
	2004-05	61st	0.408	0.331	0.365	
2010s	2011-12	68th	0.380	0.300	0.340	0.340
*Projected**						
	2022				0.350	
	2030				0.356	
	2034				0.362	
	2040†				0.358	

#National Sample Survey

$Authors' calculations from NSS data

*National Institution for Transforming India (NITI) Aayog, Government of India. https://www.niti.gov.in/sites/default/files/2021-08/Working-Group-Report-Demand-Supply-30-07-21.pdf

†Personal communication with Dr. Surabhi Mittal, International Maize and Wheat Improvement Center, South Asia, New Delhi

Supplementary Table S15. Percent bioavailability (absorption estimates) obtained through consumption of food grains for a few selected mineral element

Element	Bioavailability	Source
P	50.0	11
Ca	78.0	12
S	36.0	13
Fe	13.0	14
Zn	35.0	12
Cu	23.0	15
Mn	2.2	16
Cr	2.0	17
Si	4.0	18
Ni	15.0	19
V	1.0	20
As	89.0	21
Pb	10.0	22
Ba	20.0	23
Sr	13.0	24

Supplementary Table S16. Literature concentration range, average concentration, and optimal concentration of different mineral elements present in rice and wheat grain

Element	Crop	Literature concentration range	Average literature concentration (C_a)	Source
		mg kg^{-1} dry weight		
As	Rice	0.01-2.05	0.29	25, 26, 27, 28, 29, 30, 31, 32
	Wheat	0.002-0.089	0.026	33, 34, 35, 36, 37, 38
P	Rice	456.7-3200.0	1844.8	28, 39, 40, 41
	Wheat	311.0-6030.0	2945.5	35, 42, 43
Ca	Rice	40.0-320.0	120.0	29, 30, 39, 41, 44, 45,
	Wheat	254.2-766.7	447.0	42, 43, 46, 47
S	Rice	413.0-847.4	639.5	40, 41
	Wheat	140.0-2270.0	1426.0	33, 35, 43, 48, 49
Fe	Rice	1.0-20.0	9.0	28, 29, 30, 39, 44, 45
	Wheat	22.5-79.0	42.8	42, 43, 49, 50, 51, 52, 53, 54
Zn	Rice	3.0-27.8	16.6	28, 29, 30, 32, 39, 41
	Wheat	14.6-39.4	27.3	33, 42, 46, 47, 49, 50, 52, 53, 54, 55, 56
Mn	Rice	5.0-47.6	20.4	28, 29, 30, 32, 39, 41
	Wheat	13.0-87.0	41.2	43, 46, 47, 53, 56
Cu	Rice	0.43-7.00	3.12	28, 29, 30, 32, 44, 45
	Wheat	1.8-11.3	5.1	43, 46, 47, 49, 53, 57, 58
Si	Rice	150.0-640.0	417.8	59, 60, 61, 62, 63
	Wheat	100.0-5300.0	1012.2	64, 65, 66
Ni	Rice	0.009-2.330	0.551	28, 30, 32
	Wheat	0.13-0.62	0.28	33, 46, 50, 64
Li	Rice	0.15-0.24	0.20	67
	Wheat	NA	0.02	67
V	Rice	0.012-0.037	0.020	30, 68
	Wheat	0.002-2.700	0.625	33, 47, 66
Ba	Rice	0.013-1.200	0.424	28, 30, 69
	Wheat	0.27-13.20	5.22	35, 43, 46, 47, 50
Sr	Rice	0.014-3.700	1.102	70, 71
	Wheat	0.20-17.91	2.26	35, 43, 72, 73
Cr	Rice	0.001-0.671	0.220	28, 30, 32, 45
	Wheat	0.014-0.850	0.160	33, 51, 66, 74, 75, 76, 77, 78, 79
Pb	Rice	0.001-0.333	0.026	29, 30, 80
	Wheat	0.001-0.716	0.071	38, 47, 78, 81
Al	Rice	3.9-76.5	24.6	30, 82, 83, 84
	Wheat	0.81-50.00	19.31	43, 84, 85, 86

Supplementary Table S18. Role of mineral elements in human health systems

Element	Systems of human body												Source
	Skeletal	Muscular	Dermatological	Cardiovascular	Gastro-intestinal	Renal	Respiratory	Hematological	Nurological	Endocrine	Reproductive	Immunity	
P	+										+		87
Ca	+	+		+					+	+			88
S		+	+							+		+	89, 90
Fe		+					+	+					91, 92
Zn			+		+				+		+	+	93
Cu	+	+	+					+					94, 95
Mn						+				+			96
Si	+	+											97
Ni								+					97
V	+									+		+	20
Li									+		+		98
Ag			–										99
Ga													100
Al	–							–					101
As			–	–		–	–		–			–	97
Pb				–		–		–				–	102, 103
Cr			–		–	–	–		–	+			104
Ba				–	–	–	–				–		105, 106
Sr	–												107

+constructive effect on human health
–adverse effect on human health

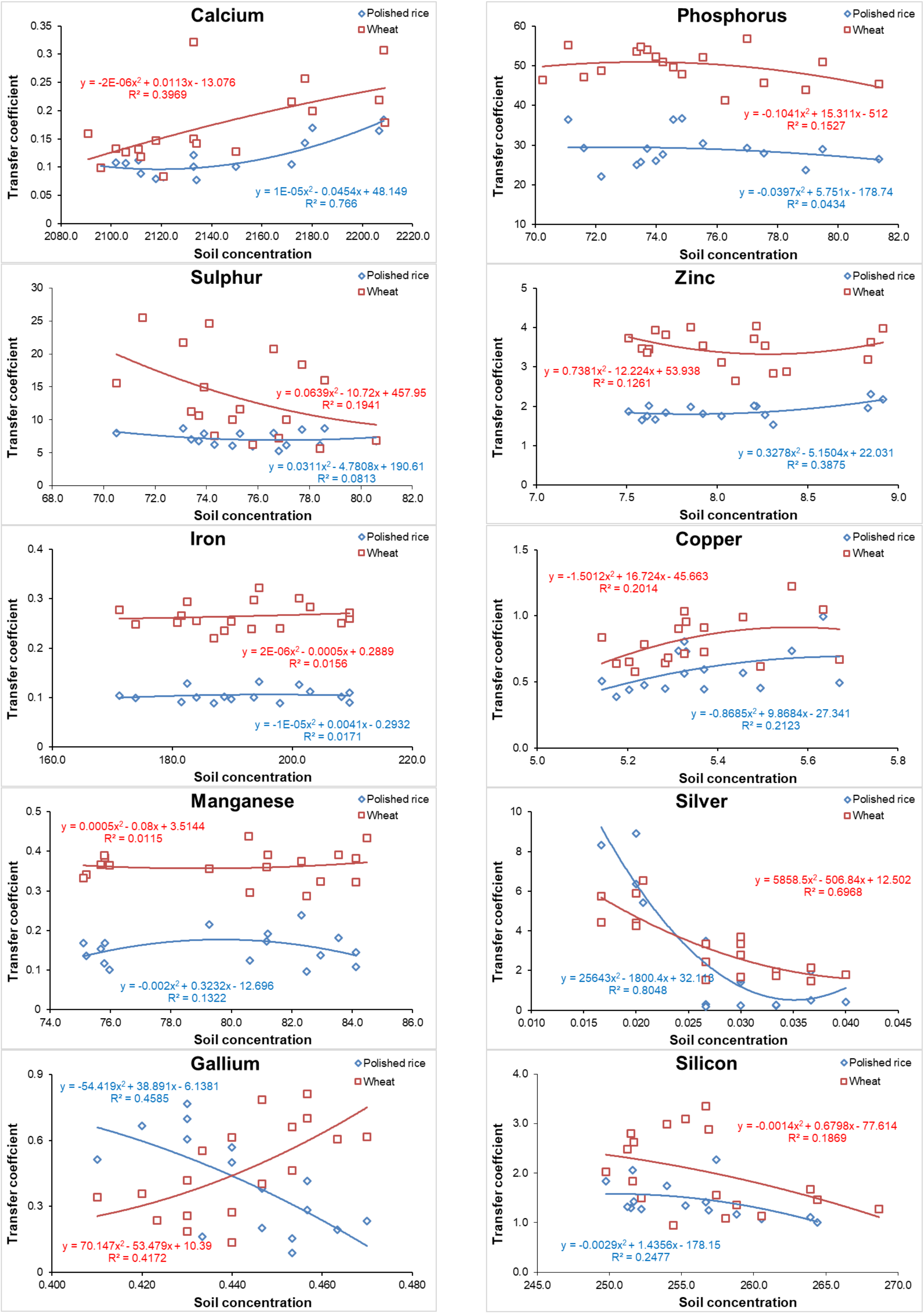

Supplementary Fig. S1. Scatter plots showing the relation between soil concentration and transfer coefficient (soil to grain) of the measured 19 mineral elements. Data are means (n = 3). Soil concentration is expressed in mg kg^{-1}.

Supplementary Fig. S1. continued

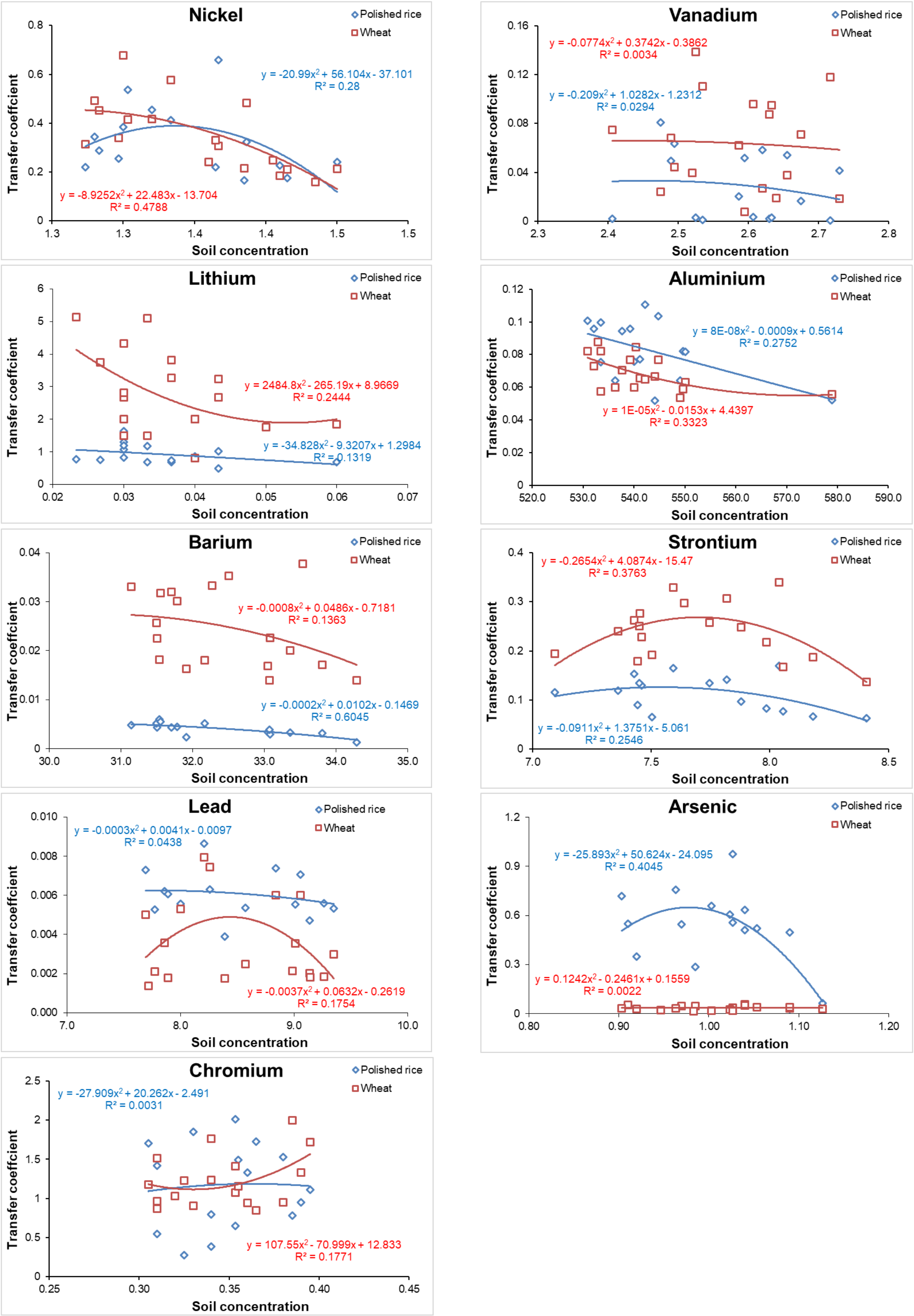

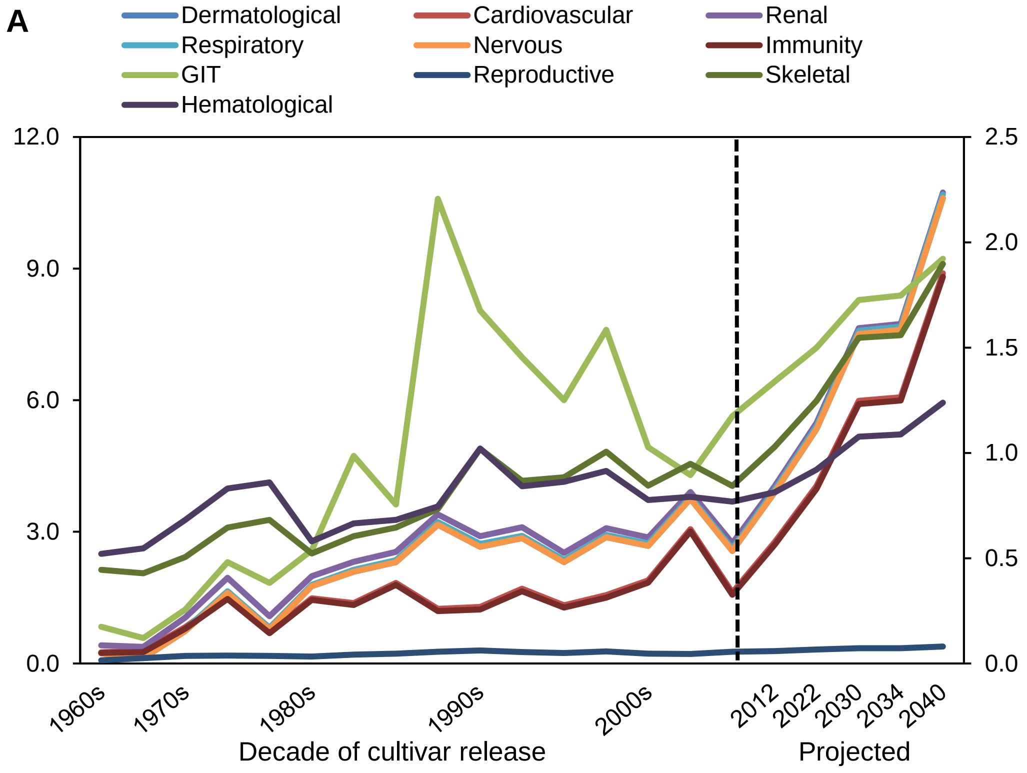

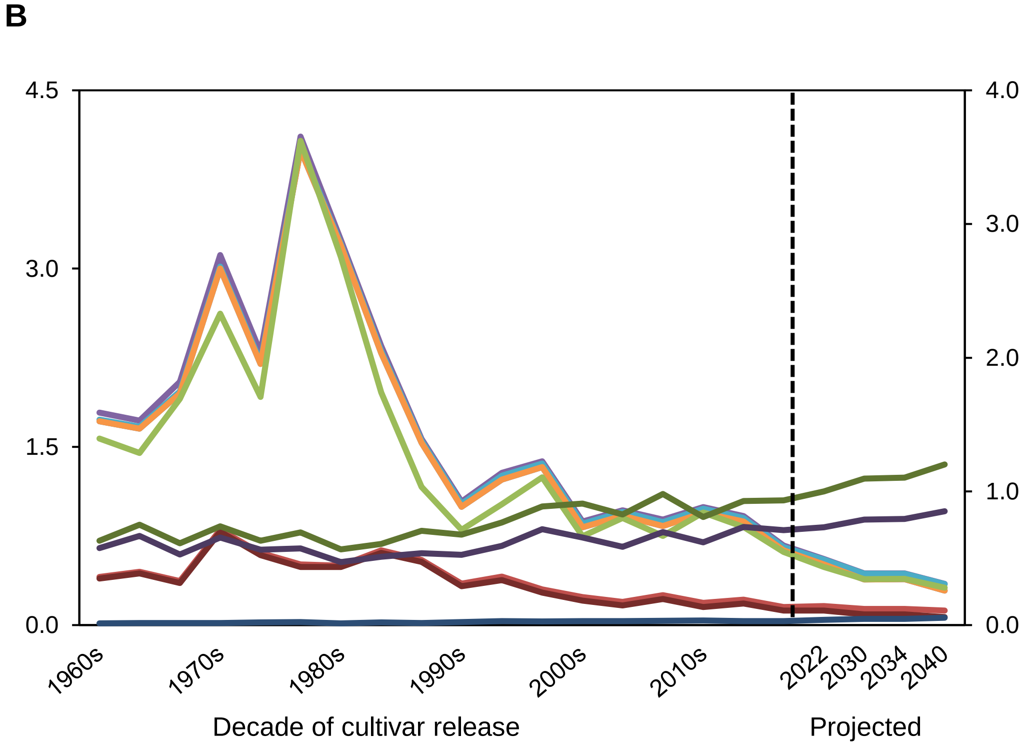

Supplementary Fig. S2. Adverse effects of mineral diet quality index (M-DQI) on different human health systems. (A) Rice. (B) Wheat. Observed data are means (*n* = 6). Horizontal lines beyond the dashed vertical line extended up to 2040 represent projected effects. Left vertical axis represents adverse effects on dermatological, cardiovascular, renal, respiratory, nervous, and immunity system; and right vertical axis represents adverse effects on gastro-intestinal, reproductive, skeletal, and haematological system.

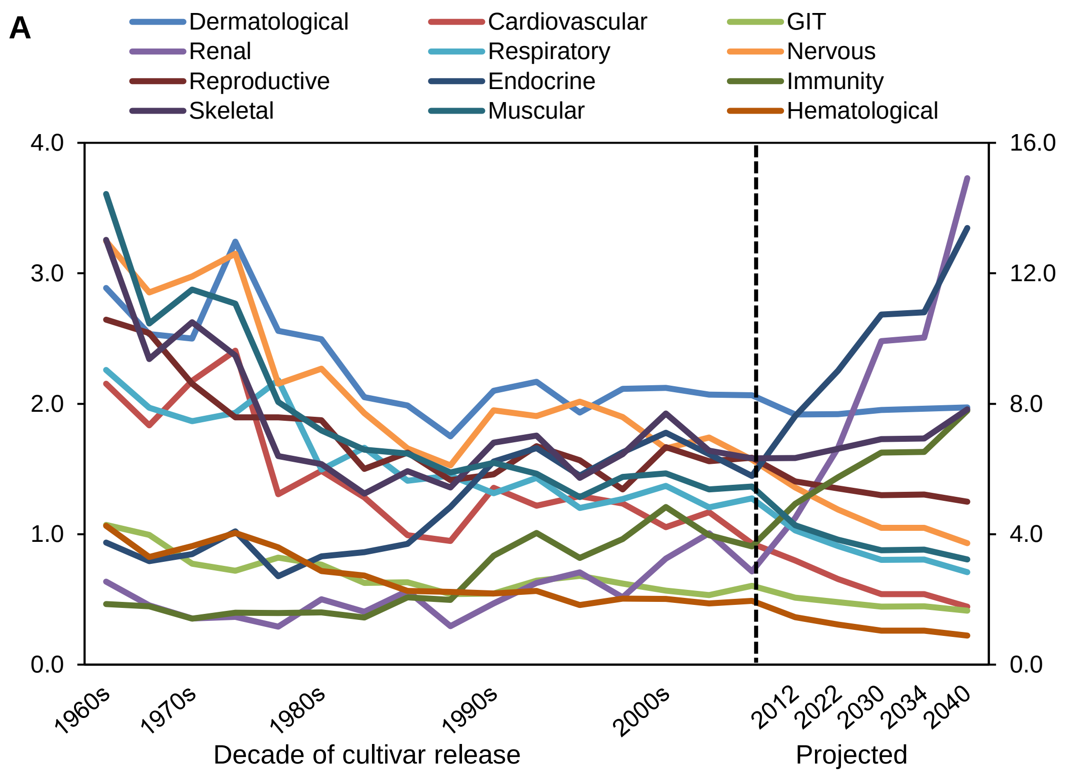

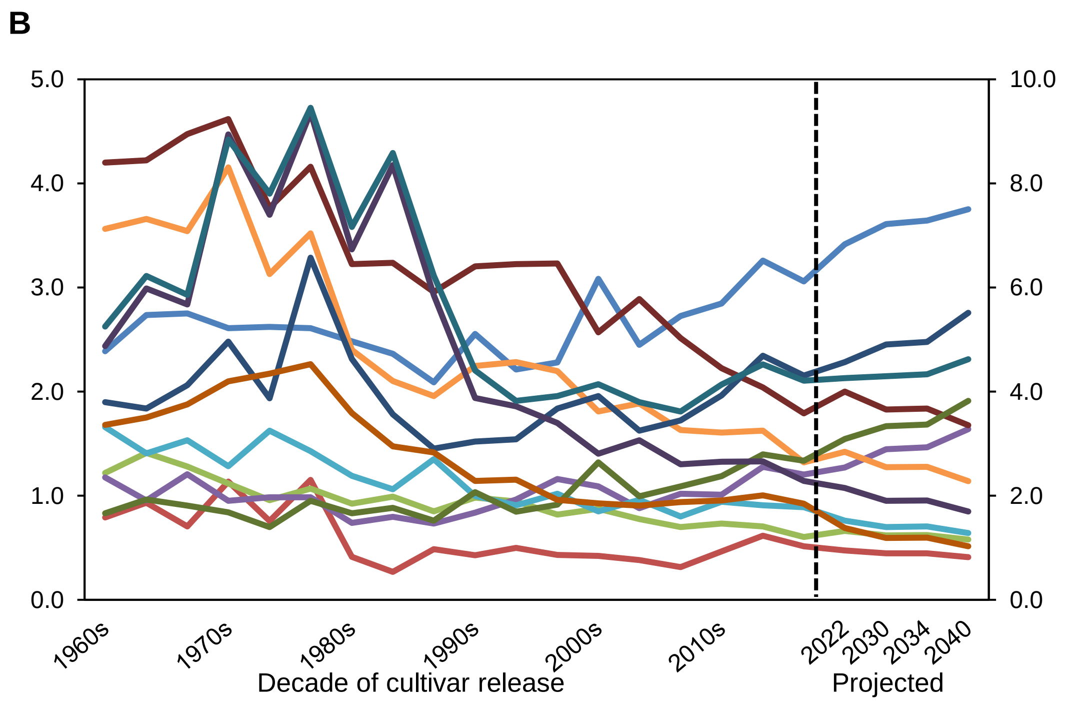

Supplementary Fig. S3. Constructive effects of mineral diet quality index (M-DQI) on different human health systems. (A) Rice. (B) Wheat. Observed data are means (*n* = 6). Horizontal lines beyond the dashed vertical line extended up to 2040 represent projected effects. Left vertical axis represents constructive effects on dermatological, cardiovascular, gastro-intestinal, renal, respiratory, nervous, and reproductive system; and right vertical axis represents constructive effects on endocrine, immunity, skeletal, muscular, and haematological system.

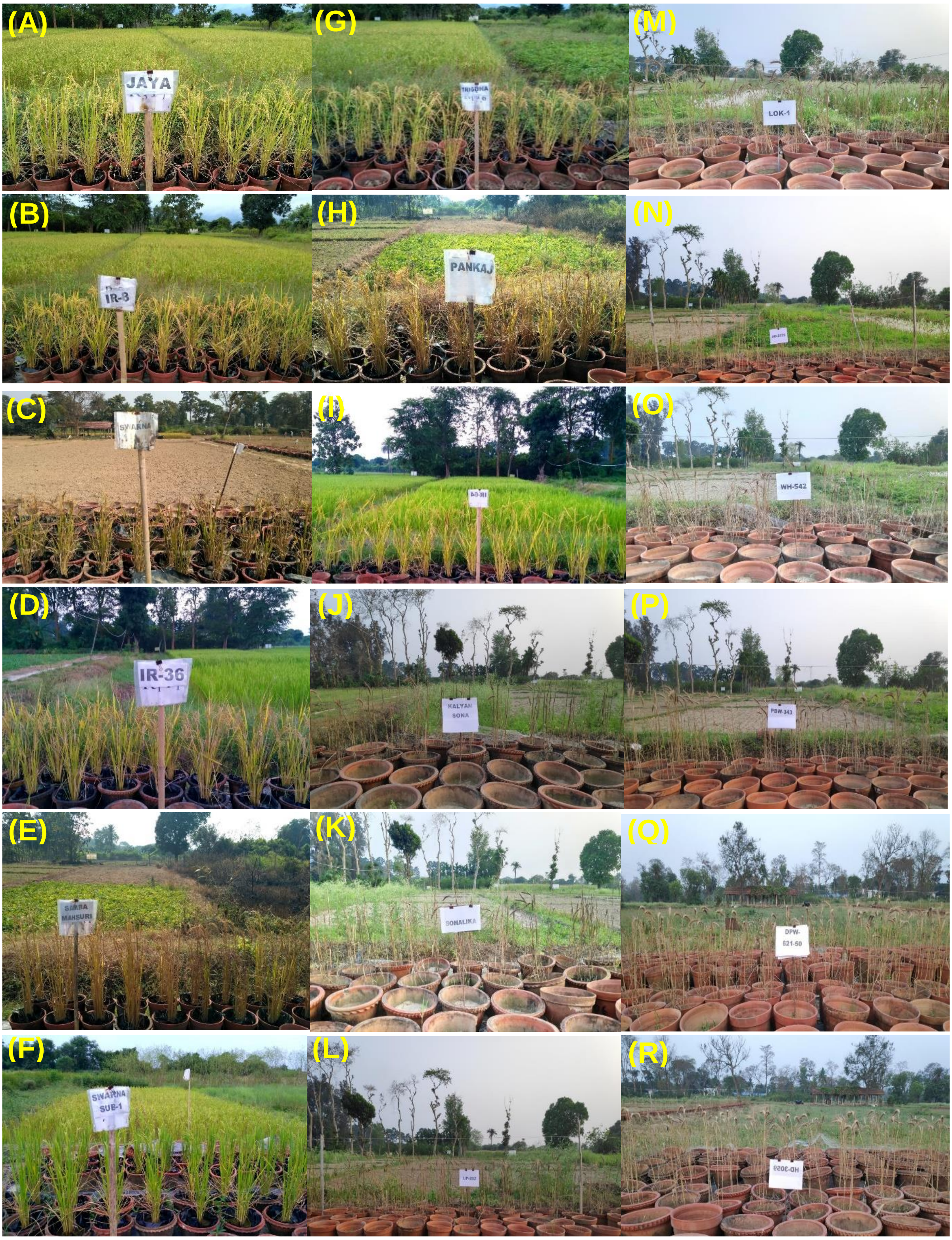

Supplementary Fig. S4**.** Experimental cultivars of rice and wheat grown in pots. Rice cultivars- (A) Jaya; (B) IR 8; (C) Swarna; (D) IR 36; (E) Samba Masuri; (F) Swarna *Sub 1*; (G) Triguna; (H) Pankaj; (I) IR 64; Wheat cultivars- (J) Kalyan Sona; (K) Sonalika; (L) UP 262; (M) LOK 1; (N) HD 2329; (O) WH 542; (P) PBW 343; (Q) DPW 621-50; (R) HD 3059

References

1. J. R. Heckman, Soil fertility test interpretation, phosphorus, potassium, magnesium, and calcium. Rutgers Cooperative Research and Extension, New Jersey Agricultural Experiment Station Rutgers, The State University of New Jersey, New Brunswick (1998).
2. J. Zbíral, Determination of plant-available micronutrients by the Mehlich 3 soil extractant –a proposal of critical values. *Plant Soil Environ.* **62**, 527–531 (2016).
3. J. Zbíral, M. Smatanová, & P. Němec, Sulphur status in agricultural soils determined using the Mehlich 3 method. *Plant Soil Environ.* **64**, 255–259 (2018).
4. C. Narayanaswamy, N. B. Prakash, Calibration and categorization of plant available silicon in rice soils of South India. *J. Plant Nutri.* **32**:1237–1254 (2009).
5. Z. V. Khanlari, and M. Jalali. Concentrations and chemical speciation of five heavy metals (Zn, Cd, Ni, Cu, and Pb) in selected agricultural calcareous soils of Hamadan Province, Western Iran. *Arch. Agron*. *Soil Sci.* **54(1)**:19-32 (2008).
6. J. G. Kim, S. S. Lee, H. S. Moon, I. M. Kang. Land application of alum sludge from water purification plant to acid mineral soil treated with acidic water. *Soil Sci. Plant Nutri.* **48**:15–22. (2002)
7. A. K. Shanker, C. Cervantes, H. Loza-Tavera, S. Avudainayagam, Chromium toxicity in plants. *Environ. Int.* **31**:739-753 (2005).
8. A. Basu, D. Saha, R. Saha, T. Ghosh, B .A. Saha, Review on sources, toxicity and remediation technologies for removing arsenic from drinking water. *Res. Chem. Intermed.***40:**447–485(2014).
9. P. R. J. Denneman, J. G. Robberse, Ecotoxicological risk assessment as a base for development of soil quality criteria. The NPO report, National Agency for the Environmental Protection, Copenhagen (1990).
10. A. Kabata-Pendias, Trace Elements in Soils and Plants. CRC Press, Boca Raton, Florida, USA (2010).
11. D. Weremko, H. Fandrejewski, T. Zebrowska, I. K. Han, J. H. Kim, W. T. Cho, Bioavailability of phosphorus in feeds of plant origin for pigs. *Asian-Australas. J. Anim. Sci***.** **10**: 551–556 (1997).
12. M. A. Levrat-Verny, C. Coudray, J. Bellanger, H. W. Lopez, C. Demigne, Y. Rayssiguier, C. Remesy, Whole wheat flour ensures higher mineral absorption and bioavailability than white wheat flour in rats. *Br. J. Nutri*. **82**:17–21 (1999).
13. T. Florin, G. Neale, G. R. Gibson, S. U. Christl, J. H., Cummings, Metabolism of dietary sulphate: absorption and excretion in humans. *Gut.* **32**: 766–773 (1991).
14. L. Hallberg, L. Hulthen, Prediction of dietary iron absorption: an algorithm for calculating absorption and bioavailability of dietary iron. *Am. J. Clin. Nutri*. **71**:1147–1160 (2000).
15. I. Egli, L. Davidsson, M. A. Juillerat, D. Barclay, R. Hurrell, Phytic acid degradation in complementary foods using phytase naturally occurring in whole grain cereals. *J. Nutri***.** **68**:1855–1859 (2004).
16. P. E. Johnson, G. I. Lykken, E. D. Korynta, Absorption and biological half-life in humans of intrinsic and extrinsic Mn tracers from food of plant origin. *J. Nutri.* **121**:711–717(1991)
17. R. A. Anderson, Chromium. In: Caballero, B. (Ed.), Encyclopedia of Human Nutrition, Academic Press, Oxford, pp. 396–401 (2005).
18. L. H. P. Jones, K. A. Handreck, The relation between the silica content of the diet and the excretion of silica by sheep. *J. Agric. Sci.* **65**:129-134 (1965).
19. WHO (World Health Organization) Nickel. Air Quality Guidelines. WHO Regional Office for Europe, Copenhagen, Denmark (2000).
20. A. R. Byrne, and L. Kosta, Vanadium in foods and in human body fluids and tissues. *Sci. Total Environ.* **10**: 17-30 (1978).
21. A. L. Juhasz, E. Smith, J. Weber, M. Rees, A. Rofe, T. Kuchel, L. Sansom, R. Naidu, In vivo assessment of arsenic bioavailability in rice and its significance for human health risk assessment. *Environ. Health Perspect*. **114**:1826–1831 (2006).
22. K. R. Mahaffey, Quantities of lead producing health effects in humans: sources and bioavailability. *Environ. Health Perspect* **19**: 285-295 (1977)

23. D. Moffett, C. Smith, Y. Stevens, L. Ingerman, S. Swarts, L. Chappell, Toxicological profile for barium and barium compounds. Agency for toxic substances and disease registry (pp. 1–231). Atlanta, Georgia: US Department of Health and Human Services (2007).
24. C. S. Marcus, F. W. Lengemann, Absorption of Ca^{45} and Sr^{85} from solid and liquid food at various levels of the alimentary tract of the rat. *J Nutri*. **77**: 155– 160 (1962).
25. S. Caroli, S. D'Ilio, M. Alessandrelli, G. Forte, S. Caroli, Arsenic content of various types of rice as determined by plasma-based techniques. *Microchem. J*. **73**, 195–201 (2002).
26. J. M. Duxbury, A. B. Mayer, J. G. Lauren, N. Hassan, Food chain aspects of arsenic contamination in Bangladesh: Effects on quality and productivity of rice. *J. Environ. Sci. Health A.* **38**, 61–69(2003).
27. I. Pizarro, M. Gómez, Evaluation of stability of arsenic species in rice. *Analytic. Bioanalytic. Chem*. **376**:102–109 (2003).
28. Z. Sha, Q. Chu, Z. Zhao, Y. Yue, l. Lu, J. Yuan, J. Y. Cao, Variations in nutrient and trace element composition of rice in an organic rice-frog co-culture system. *Sci. Rep.* **7**:15706 (2017).
29. M. Tatah Mentan, S. Nyachoti, L. Scott, N. Phan, F. O. Okwori, N. Felemban, T. R. Godebo, Toxic and essential elements in rice and other grains from the United States and other countries, *Int. J. Environ. Res. Public Health.* **17**:8128 (2020).
30. H. Tsukada, H. Hasegawa, A. Takeda, S. Hisamatsu, Concentrations of major and trace elements in polished rice and paddy soils collected in Aomori, Japan. *J. Radioanal. Nucl.* **273**:199–203 (2007).
31. P. N. Williams, A. H. Prince, A. Raab, S. A. Hossain, J. Feldmann, and A. A. Meharg, Variation in arsenic speciation and concentration in paddy rice related to dietary exposure, *Environ. Sci. Technol*. ***39***: 5531-5540 (2005)
32. B. M. Yao, P. Chen, and G. X. Sun, Distribution of elements and their correlation in bran, polished rice, and whole grain. *Food Sci. Nutri.* **8**:982-992 (2020).
33. K. Ertl, W. Goessler, Grains, whole flour, white flour, and some final goods: an elemental comparison. *Eur. Food Res. Technol.* **244**:2065–2075 (2018).
34. M. J. Gartrell, J. C. Craun, D. S. Podrebarac, E. L. Gunderson, Pesticides, selected elements, and other chemicals in adult total diet samples, October 1980 March 1982. *J. Assoc. Offic. Anal. Chem.* **69**:146–161(1986).
35. J. S. Khokhar, S. Sareen, B. S. Tyagi, G. Singh, L. Wilson, I. P. King, D. Y. Scott, M. R. Broadley, Variation in grain Zn concentration, and the grain ionome, in field-grown Indian wheat. *PLoS ONE.***13**: e0192026 (2018).
36. F. J. Zhao, F. J. Lopez-Bellido, C. W. Gray, W. R. Whalley, L. J. Clark, and S. P. McGrath, Effects of soil compaction and irrigation on the concentrations of selenium and arsenic in wheat grains. *Sci. Total Environ*. **372**:433–439 (2007).
37. F.J. Zhao, J. L. Stroud, T. Eagling, S. J. Dunham, S. P. McGrath, and P. R. Shewry, Accumulation, distribution, and speciation of arsenic in wheat grain. *Environ. Sci. Technol.* **44**:5465-5468 (2010).
38. D. Wiersma, B. J. Van Goor, N. G. Van der Veen, Cadmium, lead, mercury, and arsenic concentrations in crops and corresponding soils in the Netherlands. *J. Agric. Food Chem.* **34**:1067–1074 (1986).
39. A. S. M. Saleh, P. Wang, N. Wang, L. Yang, and Z. Xiao, Brown Rice Versus White Rice: Nutritional Quality, Potential Health Benefits, Development of Food Products, and Preservation Technologies. *Compre. Rev. Food Sci. Food Safe*. 18 (2019).
40. M. Okuda, A. Isogai, M. Joyo, N. Goto-Yamamoto, S. Mikami, Influence of sulfur and nitrogen content of rice grains on flavor in stored sake. *Cereal Chem*. **86(5)**: 534–541 (2009).
41. C. K. Reddy, L. Kimi, S. Haripriya, N. Kang, Effects of polishing on proximate composition, physico-chemical characteristics, mineral composition and antioxidant properties of pigmented rice. *Rice Sci.* **24(5)**: 241-252 (2017).
42. S. A. Mallick, K. Azaz, M. Gupta, V. Sharma, B. K. Sinha, Characterization of grain nutritional quality in wheat. *Indian J. Plant Physiol.* **18**:183–186 (2013).

43. E. Suchowilska, M. Wiwart, W. Kandler, R. Krska, A comparison of macro- and microelement concentrations in the whole grain of four *Triticum* species. *Plant Soil Environ.* **58(3)**:141–147(2012).
44. Y. Huang, C. Tong, F. Xu, Y.Chen, C. Zhang, and J. Bao, Variation in mineral elements in grains of 20 brown rice accessions in two environments. *Food Chem*. **192**: 273-278 (2016).
45. S. Huang, P. Wang, N. Yamaji, and J. F. Ma, Plant Nutrition for Human Nutrition: Hints from Rice Research and Future Perspectives. *Mol. Plant* **13**: 825–835 (2020).
46. M. R. Islam, and A. A. Meharg, Lead in Rice: Analysis of Baseline Lead Levels in Market and Field Collected Rice Grains. *Sci. Total Environ***. 485**: 428–434 (2014).
47. N. Podio, M. Baroni, R. Badini, M. Inga, H. Ostera, M. Cagnoni, E. Gautier, P. García, J. Hoogewerff, D. Wunderlin, Elemental and isotopic fingerprint of Argentinean wheat. Matching soil, water, and crop composition to differentiate provenance. *J. Agric. Food Chem.* **61**(16):3763–3773 (2013).
48. H. Zhao, B. Guo, Y. Wei, B. Zhang, S. Sun, L. Zhang, and J. Yan, Determining the geographic origin of wheat using multielement analysis and multivariate statistics. *J. Agric. Food Chem.* **59**:4397−4402. (2011).
49. H. A. Naeem, Sulfur nutrition and what quality. In: J. Jez, editor, Sulfur: A missing link between soils, crops, and nutrition. Agron. Monogr. 50. ASA, Madison, WI. p. 153–169 (2008).
50. M. S. Fan, F. J. Zhao, S. J. Fairweather-Tait, P. R. Poulton, S. J. Dunham, S. P. McGrath, Evidence of decreasing mineral density in wheat grain over the last 160 years. *J. Trace Elem. Med. Biol.* **22**:315–324 (2008).
51. K. H. Laursen, J. K. Schjoerring, J. E. Olesen, M. Askegaard, U. Halekoh, S. Husted, Multielemental fingerprinting as a tool for authentication of organic wheat, barley, faba bean, and potato. *J. Agric. Food Chem.* **59**:4385−4396 (2011).
52. H. Liu, Z. H. Wang, F. Li, K. Li, Yang, N. Yang, Y. Huang, D. Liang, H. Zhao, H. Mao, J. Liu, and W. Qiu, Grain iron and zinc concentrations of wheat and their relationships to yield in major wheat production areas in China. *Field Crop Res.* **156**: 151–160 (2014).
53. S. Saha, M. Chakraborty, D. Sarkar, K. Batabyal, B. Mandal, S. Murmu, D. Pradhan, G. C. Hazra, and R. W. Bell, Rescheduling zinc fertilization and cultivar choice improve zinc sequestration and its bioavailability in wheat grains and flour. *Field Crop Res*. **200**:10–17(2017).
54. F. Szira, I. Monostori, G. Galiba, M. Rakszegi, A. F. Bálint, Micronutrient Contents and Nutritional Values of Commercial Wheat Flours and Flours of Field-grown Wheat Varieties – A Survey in Hungary, *Cereal Res. Commun.* **42**: 293–302 (2014).
55. J. W. Wang, H. Mao, and H. B. Zhao, Different increases in maize and wheat grain zinc concentrations caused by soil and foliar applications of zinc in loess plateau, China. *Field Crop Res.* **135**:89–96 (2012).
56. M. Wang, Kong, F., Liu, R., Fan, Q. and Zhang, X. (2020). Zinc in Wheat Grain, Processing, and Food, *Front. Nutri.* 7:124
57. H. F. Gomez-Becerra, A. Yazici, L. Ozturk, H. Budak, Z. Peleg, A. Morgounov, T. Fahima, Y. Saranga, I. Cakmak, Genetic variation and environmental stability of grain mineral nutrient concentrations in *Triticum dicoccoides*under five environments. *Euphytica.* **171**:39–52 (2010).
58. A. F. Balint, G. Kovacs, L. Erdei, J. Sutka, Comparison of the Cu, Zn, Fe, Ca and Mg contents of the grains of wild, ancient and cultivated wheat species. *Cereal Res. Commun.* **29**:375–382 (2001).
59. T. H. Ansari, K. Iwasaki, T. Yoshida, Y. Yamamoto, A. Miyazaki, Status of nutrient elements in rice grain in relation to silicon accumulation pattern during grain filling. *Bangladesh Agron. J.* **19**:125-137 (2016).
60. N. Chaiwong, S. Lordkaew, N. Yimyam, B. Rerkasem, C. Prom-u-thai, Silicon nutrition and distribution in plants of different thai rice varieties. *Int. J. Agric. Biol.* **20**:669–675 (2018).
61. B. O. Juliano, ed. 1985b. Rice: chemistry and technology, 2nd ed. St Paul, MN, USA, Am. Assoc. *Cereal Chem.* 774 pp.

62. B. M. Kennedy, and M. Schelstraete, A note on silicon in rice endosperm. *Cereal Chem.* **52**: 854856 (1975).
63. C.P. Villareal, J. W. Maranville, B. O. Juliano, Nutrient content and retention during milling of brown rices from the International Rice Research Institute. *Cereal Chem*. **68**: 437-439 (1991).
64. J. E. Schultz, R. J. French, Silicon uptake by wheat and its relation to grain yield and water use. *Aust. J. Exp. Agric.* **16**:123-128 (1976)
65. S. Neu, J. Schaller, E. Dudel, Silicon availability modifies nutrient use efficiency and content, C:N:P stoichiometry, and productivity of winter wheat (*Triticum aestivum* L.). *Sci. Rep.* **7:** 40829 (2017)
66. B. White, Evaluating the effects of silicon and nitrogen fertilization on wheat production. Louisiana State University Master's Theses. 2607 (2015)
67. http://www.pharmacognosy.com.ua/index.php/vashe-zdorovoye-pitanije/zlakovyje-i-bobovyje/pshenitsa-myagkaja. Accessed on June 2022
68. D. R. Myron, S. H. Givand, and F. H. Nielsen, Vanadium content of selected foods as determined by flameless atomic absorption spectroscopy. *J. Agric. Food Chem*. **25** (2):297-300 (1977).
69. L. Ma, L. Wang, J. Tang, Z. Yang, Arsenic speciation and heavy metal distribution in polished rice grown in Guangdong province, southern China. *Food Chem*. **233**: 110–116 (2017).
70. M. Rivero-Huguet, E. Darré, Determination of total strontium in Uruguayan rice by inductively coupled plasma optical emission spectrometry (ICP-OES). *Atomic Spectro*. **27**:80-85 (2006).
71. W. Srinuttrakul, S. Yoshida, Determination of stable cesium and strontium in rice samples by inductively coupled plasma mass spectrometry. *J. Phys. Conf. Ser.* 860 012013 (2017)
72. Z. Dolijanovic, S. Roljevic Nikolic, D. Kovacevic, S. Djurdjic, R. Miodragovic, M. Jovanovic Todorovic, J. Popovic Djordjevic, Mineral profile of the winter wheat grain: effects of soil tillage systems and nitrogen fertilization. *Appl. Ecol. Environ. Res.* **17**:11757-11771 (2019).
73. V. Kovacevic, I. Kadar, M. Rastija, R. Sudar, Impacts of NPK fertilization on chemical composition of wheat grain. 48th Croatian and 8th International Symposium on Agriculture, Dubrovnik, Croatia. (2013)
74. P. Bawiec, M. Halabis, Z. Marzec, A. Kot, J. Solski, and K. Gawel, Evaluation of chromium, nickel, iron and manganese content in wheat, flour, bran and selected baked products. *Curr. Issue. Pharm. Med. Sci*. **27(2)**:71-75 (2014).
75. V. Vrček, I. VinkovićVrček, Metals in organic and conventional wheat flours determined by an optimised and validated ICP-MS method. *Int. J. Food Sci*. **47**(8):1777–1783 (2012).
76. I. Akinyele, O. Shokunbi, Concentrations of Mn, Fe, Cu, Zn, Cr, Cd, Pb, Ni in selected Nigerian tubers, legumes and cereals and estimates of the adult daily intakes. *Food Chem*. **173**:702–708 (2015).
77. G. Bermudez, R. Jasan, R. Plá, M. Pignata, Heavy metal and trace element concentrations in wheat grains: assessment of potential non-carcinogenic health hazard through their consumption. *J. Hazard Mater*. **193**:264–271 (2011).
78. M. Huang, S. Zhou, B. Sun, Q. Zhao, Heavy metals in wheat grain: assessment of potential health risk for inhabitants in Kunshan, China. *Sci. Total Environ.* **405**(1–3):54–61 (2008).
79. M. Jamali, T. Kazi, M. Arain, H. Afridi, N. Jalbani, G. Kandhro, A. Shah, J. Baig, Heavy metal accumulation in different varieties of wheat (*Triticum aestivum* L.) grown in soil amended with domestic sewage sludge. *J. Hazard Mater*. **164** (2–3):1386–1391 (2009).
80. G. J. Norton et al. Lead in rice: analysis of baseline lead levels in market and field collected rice grains. *Sci. Total Environ.* **485**: 428–434 (2014).
81. C. Ma, F. Liu, B. Hu, M. Wei, J. Zhao, H. Zhang, Quantitative analysis of lead sources in wheat tissue and grain under different lead atmospheric deposition areas. *Environ. Sci. Pollut. Res.* **26:** 36710–36719 (2019).
82. M. Parengam et al. Study of nutrients and toxic minerals in rice and legumes by instrumental neutron activation analysis and graphite furnace atomic absorption spectrophotometry. *J. Food Compos. Anal.* **23**:340–345 (2010).

83. A. Rittirong, K. Saenboonruang, Quantification of aluminum and heavy metal contents in cooked rice samples from Thailand markets using inductively coupled plasma mass spectrometry (ICP-MS) and potential health risk assessment. *Emir. J. Food Agric.* **30**: 372-380 (2018).
84. E. J. Sneddon et al. Determination of selected metals in rice and cereal by inductively coupled plasma-optical emission spectrometry (ICP-OES). *Microchem. J.* **134**:9–12 (2017)
85. I. Maksimović, R. et al. Genetic differences in aluminium accumulation in the grains of *Aegilops* and *Triticum*. *Plant Soil Environ.* **66**:351–356 (2020).
86. R. M. Norton, Wheat grain micronutrient content in southeastern Australia. 17th International Plant Nutrition Colloquium, Istanbul, Turkey (2013).
87. R. P. Heaney, Phosphorus. In: Erdman JW, Macdonald IA, Zeisel SH, eds. Present Knowledge in Nutrition. 10th ed. Washington, DC: Wiley-Blackwell pp. 447-458 (2012).
88. National Institutes of Health (NIH), Calcium fact sheet for consumers (2021).
89. S. Parcell, Sulfur in human nutrition and applications in medicine. *Sci. Rev. Altern. Med.* **7**: 22-44 (2002).
90. M. E. Nimni, B. Han, F. Cordoba, Are we getting enough sulfur in our diet? *Nutr. Metab.* **4**:24 (2007).
91. P. T. Lieu, M. Heiskala, P. A. Peterson, Y. Yang, The roles of iron in health and disease. *Mol. Aspect. Med*. **22**: 1-87 (2001).
92. T. Anand, M. Rahi, P. Sharma, G. K. Ingle, Issues in prevention of iron deficiency anaemia in India. *Nutri*. **30**: 764-770 (2014).
93. R. B. Franklin, L. C. Costello, Zinc as an anti-tumor agent in prostate cancer and in other cancers. *Arch. Biochem. Biophys.* **463**:211-217 (2007).
94. K. E. Mason, A conspectus of research on copper metabolism and requirements of man. *J. Nutr.* **109**: 1979-2066 (1979).
95. J. R. Turnlund, Human whole-body copper metabolism. *Am. J. Clin. Nutr.* **67**: 960S-964S (1998).
96. G. L. Rehnberg, J. F. Hein, S. D. Carter, R. S. Linko, J. W. Laskey, Chronic ingestion of Mn_3O_4 by rats: tissue accumulation and distribution of manganese in two generations. *J. Toxicol. Environ. Health* **9**: 175-188 (1982).
97. F. H. Nielsen, Fluoride, vanadium, nickel, arsenic, and silicon in total parenteral nutrition. *Bull. N. Y. Acad. Med.* **60**: 177-195 (1984).
98. G. N. Schrauzer, Lithium: occurrence, dietary intakes, nutritional essentiality. *Am. Coll. Nutri.* **21**: 14-21 (2002).
99. A. B. G. Lansdown, A pharmacological and toxicological profile of silver as an antimicrobial agent in medical devices. *Adv. Pharmacol. Sci.* (2010).
100. C. R. Chitambar, Gallium and its competing roles with iron in biological systems. *Biochim. Biophysic. Acta* **1863**: 2044-2053 (2016).
101. A. C. Alfrey, Aluminum. In: Merrz W, ed. Trace elements in human and animal nutrition, 5th ed, Vol.2. Orlando, FL, Academic Press. pp. 399-413 (1986).
102. H. A. Tyroler, Epidemiology of hypertension as a public health problem: an overview as background for evaluation of blood lead – blood pressure relationship. *Environ. Health Perspect.* **78**: 3-7 (1988).
103. H. L. Needleman, C. A. Gatsonis, Low level lead exposure and the IQ of children. A meta analysis of modern studies. *JAMA*. **263**: 673-678. (1990).
104. W. T. Cefalu, F. B. Hu, Role of chromium in human health and in diabetes. *Diabetes Care*. **27**: 2741-2751 (2004).
105. M. Milanov, A possibility of substituting Ca^{2+} by Ba^{2+} or Mn^{2+} during the contractile processes of complex stomach smooth muscles. *Gen. Pharmacol*. **13**: 511–513 (1982).
106. C. Polson, M. A. Green, M. R. Lee, Clinical toxicology. London: Pitman Book (1983).
107. S. P. Nielsen, The biological role of strontium. *Bone*. **35**: 583–588 (2004).
